# Centromere mechanical maturation during mammalian cell mitosis

**DOI:** 10.1038/s41467-019-09578-z

**Published:** 2019-04-15

**Authors:** Lauren A. Harasymiw, Damien Tank, Mark McClellan, Neha Panigrahy, Melissa K. Gardner

**Affiliations:** 1 Department of Genetics, Cell Biology, and Development, University of Minnesota, Minneapolis, MN 55455 USA; 2 Medical Scientist Training Program, University of Minnesota, Minneapolis, MN 55455 USA

## Abstract

During mitosis, tension develops across the centromere as a result of spindle-based forces. Metaphase tension may be critical in preventing mitotic chromosome segregation errors, however, the nature of force transmission at the centromere and the role of centromere mechanics in controlling metaphase tension remains unknown. We combined quantitative, biophysical microscopy with computational analysis to elucidate the mechanics of the centromere in unperturbed, mitotic human cells. We discovered that the mechanical stiffness of the human centromere matures during mitotic progression, which leads to amplified centromere tension specifically at metaphase. Centromere mechanical maturation is disrupted across multiple aneuploid cell lines, leading to a weak metaphase tension signal. Further, increasing deficiencies in centromere mechanical maturation are correlated with rising frequencies of lagging, merotelic chromosomes in anaphase, leading to segregation defects at telophase. Thus, we reveal a centromere maturation process that may be critical to the fidelity of chromosome segregation during mitosis.

## Abstract (web summary)

During mitosis, tension at the centromere occurs from the spindle but the role of centromere mechanics in controlling metaphase tension is poorly understood. Here, the authors report that mechanical stiffnness of the centromere matures during mitotic progression and is amplified specifically at metaphase.

## Introduction

**Fig. 1:**
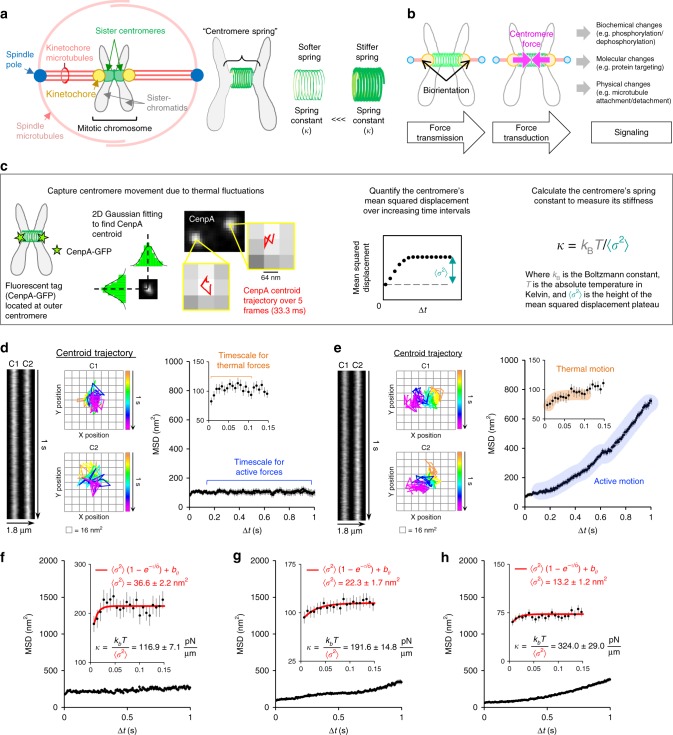
Optical assay to estimate the stiffness of the centromere-spring in human cells. **a** Each condensed mitotic chromosome (black outline, left) consists of two duplicate sister chromatids (gray, left) that are mechanically linked between the sister centromeres by the centromere-spring (green, center). The centromere-spring includes the material from the outer centromere on one sister chromatid to the outer centromere on the other (green, center). The centromere-spring’s inherent stiffness is quantified through its spring constant (right). **b** Biorientation creates a spatial separation between sister centromeres and generates centromere tension (left), which triggers biochemical, molecular, and physical changes at the centromere, kinetochore, and kinetochore microtubules (right). **c** Optical assay to measure centromere-spring stiffness. Left: Centromere movement is captured via high-resolution imaging of a fluorescent tag (CenpA-GFP) on two sister chromatids. 2D Gaussian mixture model fitting locates CenpA-GFP tags with nanometer precision, while rapid image acquisition isolates movement due to thermal fluctuations. Red trajectories show the centroid movement over the first 5 frames of 300 frames for each CenpA tag. Center: The MSD of the CenpA tag is calculated for increasing time intervals to yield the net MSD (*σ*^2^). Right: Boltzmann’s Law divides the thermal energy (numerator) by the net MSD of the CenpA tag (*σ*^2^, denominator) to calculate the spring constant of the centromere-spring (*κ*), which quantifies its stiffness. **d**, **e** Data from the Centromere Motion Tracking Protocol for two representative chromosomes. Left: Kymographs showing how CenpA-GFP tags on sister centromeres (C1, C2) change over 1 s (150 frames). Center: Trajectories for each CenpA-GFP centroid over 1 s. Right: MSD plots demonstrating the timescale for active, directed motion at longer time intervals (up to 1 s), as compared to the timescale for diffusive thermal fluctuations (inset). **f**–**h** MSD plots for three chromosomes that represent the range in derived spring constant values for the centromere-spring in RPE-1 cells. Insets: MSD data over the timescale for thermal fluctuations. The nonlinear regression fit of the MSD data at time intervals below 0.15 s to the equation for constrained motion is shown in red. All error bars represent standard error. See also Supplementary Fig. 1

During mitosis, coordinated mechanical actions between the chromosomes and the spindle are required to maintain the fidelity of chromosome segregation^1^. Within each mitotic chromosome, the centromeres of the sister chromatids play a critical role in this process (Fig. 1a, left)^2^. The centromeres of a sister-chromatid pair are mechanically linked, forming a spring-like complex, or “centromere-spring” that stretches in response to external forces (Fig. 1a, center). Here, once chromosomes become bioriented, with kinetochore microtubules originating from opposing spindle poles attached at either kinetochore (Fig. 1b, left), outwardly directed spindle forces cause the centromere spring to stretch, which generates an inwardly directed force that is commonly referred to as “tension” (Fig. 1b, center)^3^. Centromere tension has been proposed to act as a mechanical signal to the cell, broadcasting the state of chromosome-spindle attachments, and may take part in regulating the metaphase to anaphase transition^4,5^ (Fig. 1b, right). The foundation for this theory was introduced by Nicklas and Koch^6^, who used micromanipulation in grasshopper spermocytes to show that inducing tension across a detached chromosome stabilized its microtubule attachments, preventing reorientation. However, whether tension sensing is directly coupled to signaling at the kinetochore-microtubule interface remains a matter of debate^7^. Nevertheless, to determine whether tension could potentially be coupled to signaling during mitosis, it is first necessary to understand the nature of force transmission at the centromere as the cell progresses through mitosis.

Importantly, because a higher magnitude of tension is required to stretch a stiffer spring relative to a softer spring (Fig. 1a, right), it is possible that the “stiffness” of the centromere-spring plays an active role in regulating tension at metaphase, and could thus have a significant effect on chromosome segregation outcomes. While there is a robust field of work dissecting the role of the spindle and the kinetochore in integrating mechanical forces during mitosis^8,9^, and several studies have explored the contribution of centromere mechanics in directing chromosome movement during mitotic progression^10–12^, direct, quantitative measurements of centromere stiffness and tension during mitotic progression are lacking. Much of how we conceptualize centromere mechanics has evolved from studies of invertebrate systems, such as budding yeast, which contain architecturally simpler centromere systems^13^, or through the use of physically invasive methods^14,15^. In human cells, disrupting the centromere’s normal structure and function has been shown to increase the rate of chromosome missegregation^16^, a form of genome instability that is strongly implicated in cancer progression^17,18^. Thus, quantitatively elucidating the mechanical properties of the centromere in unperturbed mammalian cell systems is an important step in further dissecting the nature of tension signaling during mitotic progression and its role in genome stability^19–21^.

In this work, we develop a quantitative optical assay for measuring in vivo centromere mechanics in unperturbed, mitotic mammalian cells. Optically based approaches that preserve the cell’s normal biology provide a valuable tool to unravel the complexities of the mammalian centromere’s mechanical function. We report that the mechanical stiffness of the human centromere undergoes a maturation process during mitotic progression that follows a distinct and reproducible pattern, a process that we refer to as “centromere mechanical maturation”. Importantly, this maturation results in amplified centromere tension in metaphase. Further, we find that impaired centromere mechanical maturation in aneuploid cancer and non-cancer cells leads to decreased tension at metaphase. Thus, our results reveal a role for centromere mechanical maturation in regulating metaphase tension during mitosis, which may be critical to the fidelity of chromosome segregation during mitosis.

## Results

### Estimating centromere-spring stiffness in human cells

Through optical motion tracking of a fluorescently tagged protein incorporated into a cellular filament such as mitotic chromatin, it is possible to derive physical properties of the filament itself. In the case of the centromere-spring, this includes its spring constant (*κ*; Fig. 1a, right), which is a quantitative measure of the spring’s inherent stiffness^22–24^. When optical motion tracking of the tagged protein is applied with high spatial and temporal resolution (Fig. 1c, left), its mean squared displacement (MSD) due to thermal fluctuations in the system can be quantified. For a protein incorporated at the centromere and thus subject to the constraint of the centromere-spring, the MSD data obtained at time frames reflecting thermal fluctuations display a pattern characteristic of constrained diffusive motion, in which there is a rapid rise in displacement followed by plateau, the height of which reflects the degree of the spring’s constraint under thermal forces (Fig. 1c, center). Boltzmann’s Law, a fundamental physical law that relates the movement of a particle to the thermal energy present in the system, can then be applied to calculate the spring’s stiffness, as quantified by its spring constant (Fig. 1c, right, equation)^25^. We recently reported an optical method for estimating centromere-spring stiffness in mitotic budding yeast cells in this manner^26^.

To evaluate centromere-spring stiffness in mammalian cells, we used human hTERT-RPE-1 cells that expressed a fluorescent marker (CenpA-GFP) at the outer centromere of each chromatid (Fig. 1c, left). Since our application of Boltzmann’s law to estimate centromere-spring stiffness requires visualizing the centromere’s diffusive thermal motion rather than its random or directed motion in response to active forces such as molecular motors and microtubule dynamics, we used an optical protocol designed to capture the centromere’s position at a timescale that reflects thermal motion (Fig. 1d, e, left and center; see Methods). After applying our optical protocol to mitotic RPE-1 cells, we quantified the MSD of an individual CenpA-GFP tag for time intervals up to 1 s (Fig. 1d, e, right; see Methods). As expected, at longer time lapse intervals, the centromere’s movement showed varying degrees of motion derived from active forces, which reflects the range in movement that chromosomes undergo during mitosis (Fig. 1d, e, right). However, at time intervals up to 0.1–0.2 s, there was a reproducible pattern of constrained diffusive motion (Fig. 1d, e, insets on right). These results confirmed our assay’s ability to capture the constrained thermal motion of the centromere in mammalian cells proceeding normally through mitosis. Finally, we fit the MSD plots obtained from imaging sister centromeres to an equation for constrained diffusive motion in order to estimate the spring constant of the chromosome’s centromere-spring (Fig. 1c, right; see Methods).

For individual chromosomes, we derived centromere-spring constant values ($\kappa _{{\mathrm{{RPE}}} - 1}$) of ~100–400 pN/μm (Fig. 1f–h). These estimates are substantially higher than our finding for budding yeast ($\kappa _{{\mathrm{{yeast}}}}$ ~ 16 pN/μm), likely because budding yeast cells contain an architecturally simpler point centromere^26^. This is consistent with a reported estimate for the spring constant of the centromere in fission yeast (~42 pN/μm), which was obtained through laser ablation experiments^27^, as centromeres in fission yeast fall between budding yeast and human centromeres in terms of complexity^28^. Our stiffness estimate for the spring constant of the centromere-spring is also within range of the stiffness estimates from related chromosome structures that were reported from experiments using direct, physical manipulation. For example, in vitro microneedle manipulations have yielded spring constants of ~100 pN/μm for the arm of chromosomes from mitotic newt cells^29^. Similarly, Cojoc and colleagues used laser microsurgery to estimate the spring constant of the kinetochore in mammalian PtK1 cells at ~1000 pN/μm^30^, which is consistent with the kinetochore being substantially stiffer than the centromere due to its purely protein-based construction^31,32^.

### Centromere-spring maturation during mitotic progression

Over the course of mitosis, there is significant plasticity in the chromosomes’ structure as they progressively condense and the sister chromatids resolve^33^. Intuitively then, it may be expected that chromosome mechanical properties are similarly dynamic during this period. To test the idea that the mechanics of the centromere-spring are altered during normal mitotic progression, we quantified the stiffness of the centromere-spring at early-prometaphase, late-prometaphase, and metaphase in unperturbed RPE-1 cells.

**Fig. 2:**
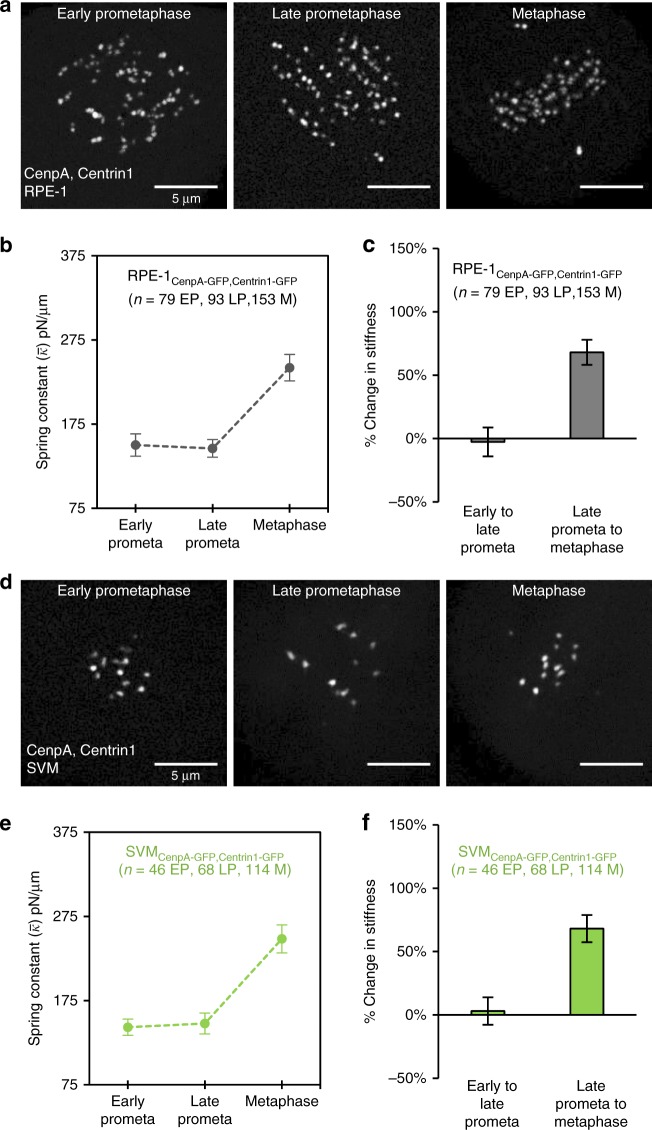
Stiffness of the centromere-spring matures during mitotic progression. **a** Live cell imaging of human RPE-1 cells expressing CenpA-GFP at the outer centromere and Centrin1-GFP at the spindle poles. Images are maximum intensity projections from full-cell-volume image series, and are representative of the described mitotic stages: early-prometaphase (left), late-prometaphase (center), and metaphase (right). **b** Mean spring constant by mitotic stage for RPE-1 cells (early-prometaphase = 79 chromosomes, 39 cells; late-prometaphase = 93 chromosomes, 26 cells; metaphase = 153 chromosomes, 49 cells). **c** Percent change in mean stiffness of the centromere-spring between early- and late-prometaphase (left) and late-prometaphase and metaphase (right) for RPE-1 cells. Percentages calculated using data shown in 2B. **d** Live cell imaging of Indian muntjac SVM cells expressing CenpA-GFP at the outer centromere and Centrin1-GFP at the spindle poles. Images as described in **a**. **e** Mean spring constant by mitotic stage for SVM cells. **f** Percent change in mean stiffness of the centromere-spring between early- and late-prometaphase (left) and late-prometaphase and metaphase (right) for SVM cells. Percentages calculated using data shown in **e**. The scale bar for all images is 5 μm. All error bars represent standard error. All *n*-values listed are chromosome numbers. See also Supplementary Figs. 1 and 6

We defined early-prometaphase cells as those in which the nuclear envelope was absent and the centromere pairs were distributed in a ring around the central spindle axis (Fig. 2a, left), a spatial arrangement that is specific to this time point^34,35^. Late-prometaphase cells were defined by the presence of parallel tracks of centromere pairs migrating toward the cell equator (Fig. 2a, middle). At this stage, the centrosomes had also relocated from the cell’s center to opposing points in the periphery. Cells were considered to be at metaphase when all centromere pairs were tightly clustered at the cell equator and aligned along the metaphase plate (Fig. 2a, right). We excluded cells in which one or more chromosomes remained located in the cell periphery in the presence of a clearly formed metaphase plate, as this phenotype was seen in less than 5% of cells (Supplementary Fig. 1B), and it was unknown if chromosomes at this point more accurately reflected conditions present in late-prometaphase or during metaphase.

For each time point, we pooled data from individual chromosomes across multiple cells in order to estimate the stage-specific stiffness of the centromere (Fig. 2b). For early- and late-prometaphase chromosomes, the mean spring constant for the centromere-spring remained constant at 150.2 ± 13.2 pN/μm (mean ± SEM) and 146.2 ± 10.5 pN/μm, respectively (Fig. 2b, c). In contrast, the mean spring constant increased substantially at metaphase (242.1 ± 15.7 pN/μm): there was a 66% increase in the stiffness of the spring constant from late-prometaphase to metaphase. Thus, while we observed the centromere-spring’s stiffness to be constant from early- to late-prometaphase, stiffness increased significantly in a step-like fashion between late-prometaphase and metaphase (Fig. 2b, c).

Chromatin’s complex mechanical properties are in part due to the packaging of DNA around nucleosomes^36,37^. In cells expressing both a fluorescent fusion protein and the native protein from the endogenous loci, the protein’s total cellular concentration may rise significantly above endogenous levels. Thus, we next considered whether over-expression of CenpA-GFP influenced centromere stiffness. We repeated our experiments in an RPE-1 cell line that expressed CenpA-GFP at a near-endogenous level^38^. In this cell line (Supplementary Fig. 1C), cellular CenpA levels, as measured by quantitative western blotting, were nearly five times lower than in the over-expressing line (Supplementary Fig. 1D, E). Regardless, we obtained comparable results for both the absolute spring constant estimates and the relative change in stiffness across mitotic stages between the two cell lines (Supplementary Fig. 1F, G). Therefore, we concluded that the pattern of stiffness change we observed during mitosis was not an artifact of CenpA over-expression.

### Conservation of centromere-spring stiffness maturation

We next asked whether this pattern of stiffness change was conserved in a non-human mammalian species. To answer this question, we repeated our experiments in an SV40-transformed Indian muntjac (*Muntiacus muntjak*) cell line (SVM). Indian muntjac cells proceed through mitosis in stages qualitatively equivalent to those observed for RPE-1 cells (Fig. 2d), but have a diploid chromosome number of 6–7, depending on gender, and the chromosomes are significantly larger than human chromosomes^39^. Consistent with our findings in human cells, the stiffness of the muntjac centromere-spring also increased in a stage-specific manner during mitosis. At early- and late-prometaphase, the estimated spring constant for the muntjac centromere-spring was 143.3 ± 9.7 and 144.0 ± 11.6 pN/μm, respectively (Fig. 2e, f). At metaphase, stiffness increased by 72% to 248.3 ± 16.7 pN/μm. Thus, we conclude that there is a signature maturation pattern in centromere stiffness during mitosis that is conserved across a human and non-human mammalian species. The reproducibility of these results, even in the presence of species-specific differences in chromosome architecture and number, strongly suggests that a stage-specific increase in centromere mechanical stiffness is a foundational principle of chromosome function during mitosis.

### Effect of kinetochore-microtubule attachment and dynamics

**Fig. 3:**
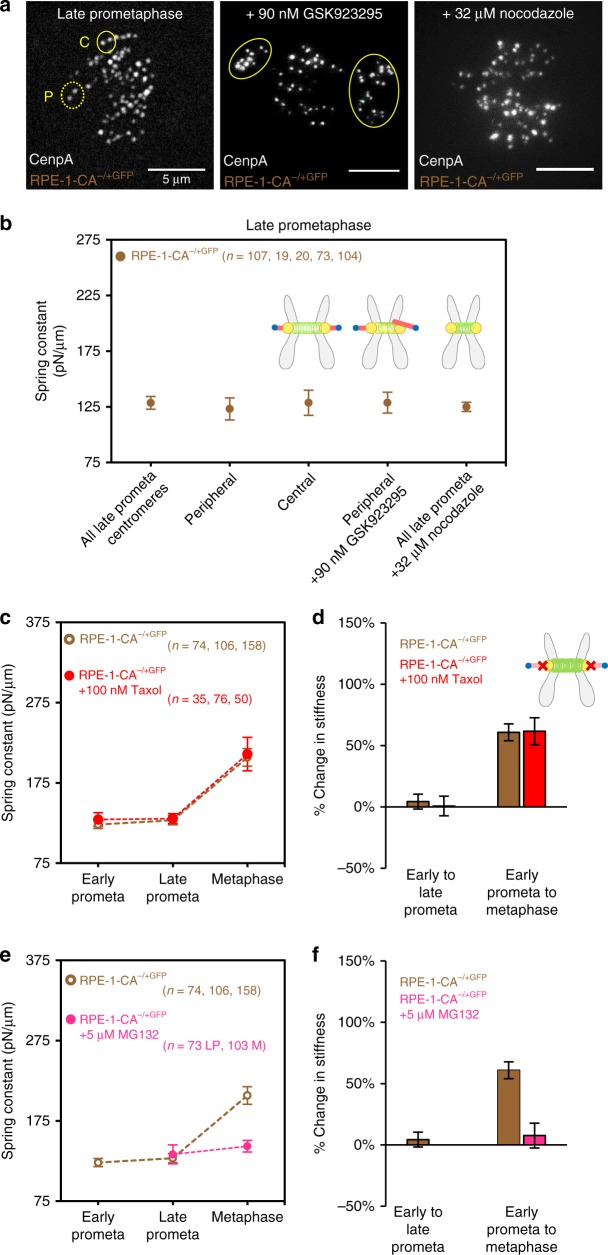
Centromere-spring matures independently of microtubule attachments. **a** Live cell imaging of an untreated late-prometaphase RPE-1 (left) showing a peripheral chromosome (dashed yellow circle, P) as compared to a central chromosome (solid yellow line, C). Compare with (center) a cell treated with 90 nM GSK-923295 and (right) 32 μM Nocodazole. Treatment with GSK-923295, a rigor inhibitor of CenpE, creates a population of chromosomes with lateral attachments that remain trapped at the spindle poles (yellow ovals). Treatment with high-dose Nocodazole depolymerizes kinetochore-microtubule attachments, causing chromosomes to disperse throughout the cell. **b** Mean spring constant for late-prometaphase chromosomes under varying conditions. From left to right: all prometaphase chromosomes, only central chromosomes, only peripheral chromosomes, laterally attached chromosomes (GSK-923295 treated), and unattached chromosomes (Nocodazole treated). **c**, **d** Mean spring constant by mitotic stage for RPE-1-CA^−/+GFP^ cells treated with 100 nM Taxol (**c**, red filled data points), and the percent change in the mean centromere-spring’s stiffness across mitotic stages (**d**, red bars; all *n*-values as in panel **c**). Data from untreated RPE-1-CA^−/+GFP^ cells included for comparison (**c**, brown unfilled data points; **f**, brown bars). Note that both the absolute spring constant estimates and the relative change in stiffness are indistinguishable between the two cell types. **e**, **f** Mean spring constant by mitotic stage for RPE-1-CA^−/+GFP^ cells treated with 5 μM MG132 (**c**, pink filled data points), and the percent change in the mean centromere-spring’s stiffness across mitotic stages (**d**, pink bars; all *n*-values as in panel **c**). Data from untreated RPE-1-CA^−/+GFP^ cells included for comparison (**c**, brown unfilled data points; **f**, brown bars). No estimate for early-prometaphase is included for MG132-treated chromosomes, as cells were exposed during prophase and early-prometaphase. All error bars represent standard error. All *n*-values listed are chromosome numbers

We next assessed whether the chromosome’s kinetochore-microtubule attachment type, either lateral or end-on, contributed to our centromere-spring stiffness measurements. We targeted a plus end-directed kinesin (CenpE) that carries laterally attached kinetochores from the spindle poles to the cell’s equator, thus allowing for the generation of end-on attachments at the equator. Thus, we used a rigor inhibitor of CenpE (GSK-923295) to generate populations of pole-proximal centromeres in late-prometaphase with at least one lateral-attachment (Fig. 3a, center, yellow circles)^40^. After treating cells with the CenpE inhibitor, we found no difference in mean centromere-spring stiffness values between the centromeres with laterally attached kinetochores that were proximal to the spindle poles, and the centromeres that were located at the forming metaphase plate (Fig. 3b; *z* = 4E-4, *p* > 0.99), indicating that the conversion from lateral to end-on attachments, and the position within the spindle, does not significantly impact centromere mechanical maturation.

Next, we explored whether centromere-spring stiffness was influenced by the chromosome position within the spindle, which accounts for changes in polar ejection forces that are higher in magnitude at the poles and lower in magnitude near the spindle equator^41^. Thus, we compared centromeres at late-prometaphase that were situated near the periphery (Fig. 3a, left, yellow dashed circle) with those that had arrived at the spindle equator (Fig. 3a, left, yellow solid circle). We found that there was no significant difference in the magnitude of the centromere-spring stiffness between the pole-proximal and central centromeres (Fig. 3b; *z* = 0.02, *z*-test, *p* = 0.99).

**Fig. 4:**
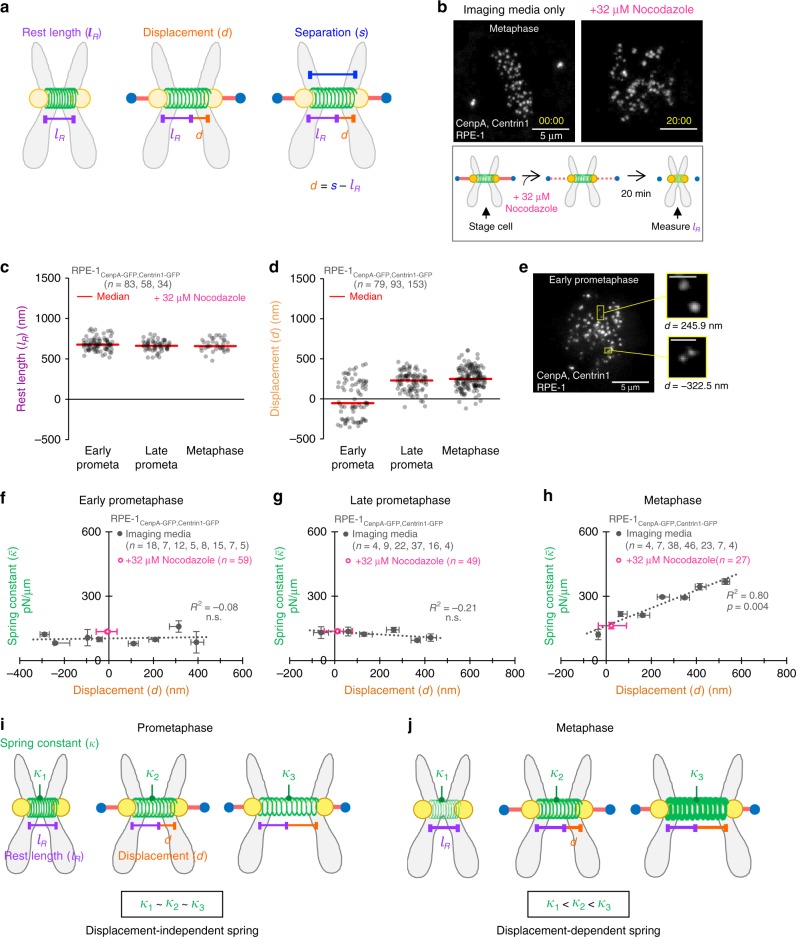
Centromere-spring stiffness is displacement-dependent at metaphase. **a** Schematic representation of the relationship between the rest length of the centromere-spring ($l_R$); centromere-spring displacement (*d*); and sister centromere separation (*s*). **b** Top: Live cell imaging of a metaphase RPE-1 cell before (left) and 20 min after (right) addition of nocodazole to a final concentration of 32 μM. Scale bar, 5 μm. Bottom: graphical representation of experimental sequence. **c** Quantification of the rest length of the centromere-spring ($l_R$), by mitotic stage for RPE-1 cells. Data points reflect individual chromosome data points pooled across three independent experiments; the group median is shown in red. **d** Quantification of the centromere-spring’s displacement by mitotic stage for each of the RPE-1 chromosomes used in the spring constant analysis (Fig. 2b). Data points reflect individual chromosomes; the group median is shown in red. **e** Left: Live cell imaging of an early-prometaphase RPE-1 cell. Scale bar, 5 μm. Right: Sister centromere pairs demonstrating the range in centromere displacement values for chromosomes in a single cell at early-prometaphase. Scale bars, 1 μm. **f**–**h** The relationship between the centromere-spring displacement and its spring constant at early-prometaphase (**f**), late-prometaphase (**g**), and metaphase (**h**) for RPE-1 cells. Chromosomes were subgrouped by displacement in 100 nm intervals starting at the group minimum. Only subgroups with four or more chromosomes are shown. Each data point reflects the subgroup’s median displacement and mean spring constant; *X*-axis error bars represent IQR; *Y*-axis error bars represent standard error. The least-squares regression fit is indicated by the dotted line. Exact *p* values from linear regression fit are shown for models meeting statistical significance; all others are indicated as non-significant (n.s.). Data for the nocodazole-treated metaphase chromosomes are shown (**g**, magenta data point), but not included in the regression fit. **i**, **j** Model illustrating the relationship between displacement of the centromere-spring and its stiffness during mitotic progression. During early- and late-prometaphase (**i**), the stiffness of the centromere-spring is displacement-independent. At metaphase (**j**), the stiffness of the centromere-spring becomes displacement-dependent. All *n*-values listed are chromosome numbers

To determine whether the presence of kinetochore-microtubule attachments themselves impacted our centromere-spring stiffness measurements, such that the magnitude of centromere stiffness would be altered in the absence of kinetochore microtubules, we exposed cells to a high-dose Nocodazole treatment (Fig. 3a, right), and then compared the magnitude of the centromere-spring stiffness in late-prometaphase between treated and untreated cells. When comparing the Nocodazole-treated cells to untreated cells, we found no difference in mean centromere-spring stiffness (Fig. 3b; *z* = 0.01, *z*-test, *p* > 0.99; similar to Nocodazole results in metaphase (see Fig. 4f–h, below)).

Finally, we tested whether kinetochore-microtubule dynamics influenced the magnitude of our centromere-spring stiffness measurements. Thus, we exposed cells to Taxol, which stabilizes spindle microtubule dynamics. We then measured the stiffness of the centromere-spring at early-prometaphase, late-prometaphase, and metaphase to account for possible differences in microtubule dynamicity in each stage. However, in all cases, we found that the centromere’s spring constant was nearly identical to that of untreated chromosomes (Fig. 3c), and, thus, the maturation pattern we described previously was unaffected (Fig. 3d; *z* = 0.07, *z*-test, *p* = 0.95).

In contrast, by preventing proper protein degradation during mitosis with MG132 treatment, we found that centromere maturation between late-prometaphase and metaphase was suppressed (Fig. 3e, f; *z* = 12.5, *p* < 8E-33), suggesting that centromere mechanical maturation, similar to other dynamic mitotic processes, may be under cell cycle regulatory control.

### Centromere maturation and sister centromere separation

Following nuclear envelope breakdown at the onset of mitosis, sister centromeres are progressively separated, stretching the centromere-spring, as the bioriented kinetochores make increasingly stable connections with a growing number of kinetochore microtubules (Fig. 1b). Thus, we next evaluated whether the increase in centromere stiffness at metaphase that we observed in the RPE-1 cells could be caused by increased stretching of the centromere-spring.

When the centromere-spring stretches in response to forces from the mitotic spindle, it increases the total sister centromere separation distance (*s*; Fig. 4a, right). This distance represents the sum of the centromere-spring rest length ($l_R$; Fig. 4a, left), which is the length of the centromere-spring in the absence of external forces, plus the added displacement induced by spindle forces (*d*; Fig. 4a, middle). To directly evaluate displacement of the centromere-spring (*d*; Fig. 4a, middle) as a function of mitotic stage, we first measured its mean rest length ($l_R$; Fig. 4a, left) at each mitotic stage by treating cells with high-dose Nocodazole (Fig. 4b, bottom). This treatment depolymerized the kinetochore-microtubule attachments, causing the centromere-spring to relax to its rest length (Fig. 4b, top). We found that the centromere-spring rest length was similar regardless of mitotic stage (Fig. 4c).

Next, we determined the displacement of the centromere-spring (*d*) by subtracting the stage-specific, mean rest length ($l_R$), as described above, from the total sister centromere separation distance (*s*) measured for each individual chromosome (Fig. 4a, right-bottom). We found that centromere-spring displacement at early-prometaphase was wide ranging (median = −46.4 nm, interquartile range (IQR) = 458.1 nm; Fig. 4d, e). This is not entirely unexpected, given that at early-prometaphase the sister chromatids may not yet be fully resolved, the mitotic spindle is incompletely formed, and kinetochore-microtubule attachments are newly forming and subject to a high turnover rate^33,35,42,43^. However, by late-prometaphase, there was a significant increase in the centromere-spring’s displacement (median = 232.8 nm, KW_Stat_ = 34.5, one-way Kruskal–Wallis, *p* = 2*e*−9; Fig. 4d). The displacement of the spring was also more uniform at late-prometaphase (IQR = 163.7 nm, BF_Stat_ = 56.1, Brown–Forsythe test, *p* = 3*e*−12), with the majority of chromosomes positively displaced (88/93, 95%). These differences likely reflect a larger percentage of chromosomes becoming bioriented and thus experiencing a net poleward force from the mitotic spindle. However, surprisingly, displacement of the centromere-spring did not continue to increase significantly from late-prometaphase to metaphase (median = 251.1 nm, KW_Stat_ = 1.7, one-way Kruskal–Wallis, *p* = 0.20; Fig. 4d), and there was no significant change in the variability of displacement (IQR = 179.7 nm, Brown–Forsythe test, BF_Stat_ = 0.47, *p* = 0.49). Thus, in direct contrast to our estimates of the centromere-spring stiffness, which increased sharply at metaphase (Fig. 2c), displacement of the centromere-spring did not increase at metaphase, but rather leveled off prior to reaching metaphase. We conclude that the stiffness of the centromere-spring matures independently of changes in the separation of the sister centromeres during mitotic progression.

### Centromere stiffness is displacement-dependent in metaphase

The simplest model for the mechanics of the centromere-spring proposes that as the spring is extended above its rest length there is no change in its stiffness. However, it is also possible that the stiffness of the centromere-spring could increase based on the magnitude of its displacement. If the centromere-spring stiffness is displacement-dependent, it would become progressively stiffer as it is increasingly displaced from its rest length. Thus, we next investigated whether the centromere-spring functioned as displacement-dependent spring in early- and late-prometaphase, and at metaphase.

At both early- and late-prometaphase, the stiffness of the centromere-spring was indistinguishable across the range of observed displacement values (Fig. 4f, g). However, in striking contrast, at metaphase, the stiffness of the centromere-spring increased substantially as a function of its displacement (Fig. 4h). For example, the centromere-spring was ~170% stiffer when the stretching displacement was 500–600 nm as compared to 0–100 nm (Fig. 4h).

These results describe a model in which the relationship between the displacement of the centromere-spring and its stiffness depends on the temporal stage of the mitotic chromosome. Specifically, during prometaphase, the centromere-spring operates as a displacement-independent spring, in which stiffness remains constant regardless of the degree to which the centromere-spring is stretched from its rest length (Fig. 4i). However, at metaphase, the centromere-spring transitions to a nonlinear, displacement-dependent spring, where stiffness increases significantly as a function of centromere-spring’s displacement (Fig. 4j).

### Displacement-dependent centromere-spring amplifies tension in metaphase

**Fig. 5:**
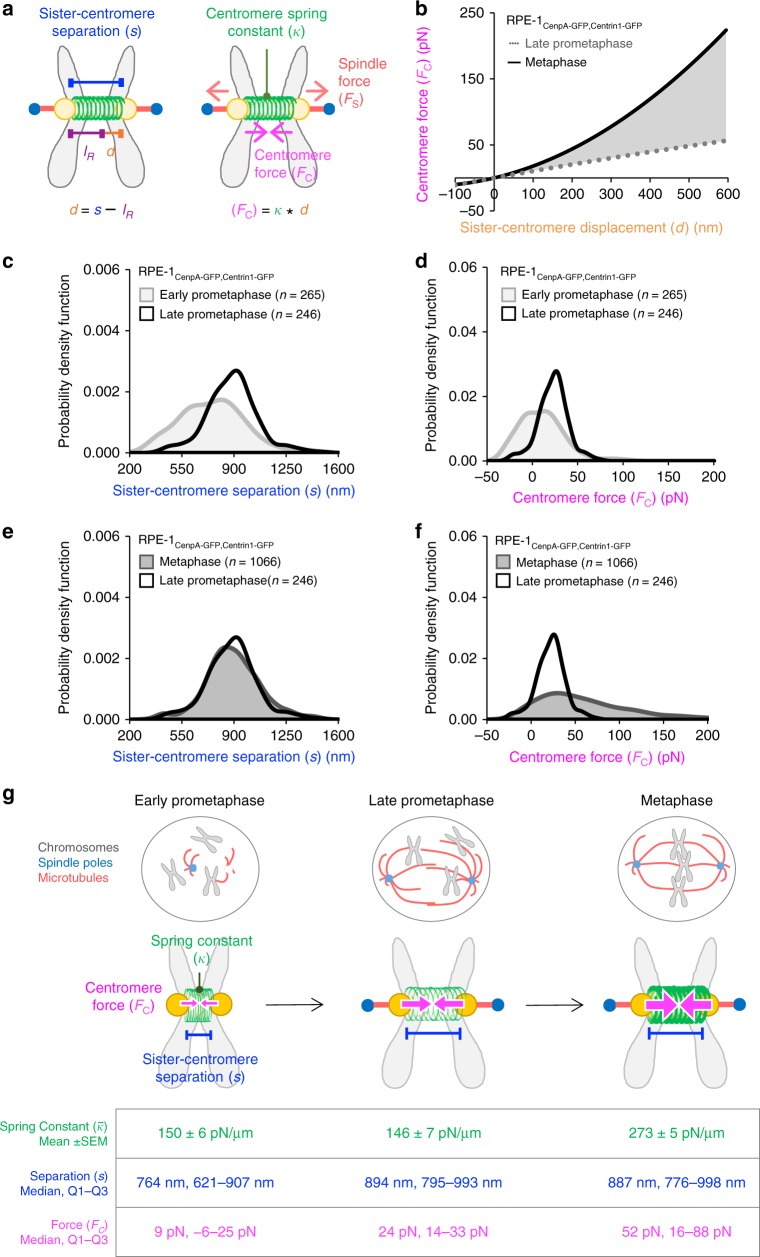
Displacement-dependent centromere-spring amplifies metaphase tension. **a** Schematic representation of centromere force during mitosis. During mitosis, the distance separating sister centromeres (*s*) is equal to their rest length $\left( {l_R} \right)$ plus the displacement (*d*) created by the external spindle force (*F*_S_). Displacement of the sister centromeres creates an internal, inward-directed centromere force *F*_C_. **b** The dynamic range in force transmission for a late-prometaphase chromosome (dotted gray line) versus a metaphase chromosome (solid black line) in RPE-1 cells. Centromere force at each stage was calculated using the displacement-specific spring constant (Fig. 4e–g) over the range of observed displacement values. The shaded region reflects the increase in dynamic range (+250.7%) between late-prometaphase and metaphase. **c**, **d** Probability density functions for individual observations of **c** sister centromere separation (*s*) and **d** centromere force (*F*_C_) at early-prometaphase (light gray line, shaded area) and late-prometaphase (black line) for RPE-1 cells (early-prometaphase: chromosomes = 265, cells = 44; late-prometaphase: chromosomes = 246, cells = 47; metaphase: chromosomes = 1066, cells = 95). **e**, **f** Probability density functions for individual observations of **e** sister centromere separation (*s*) and **f** centromere force (*F*_C_) at late-prometaphase (black line) and metaphase (dark gray line, shaded area) for RPE-1 cells. **g** Schematic illustrating centromere mechanical maturation. Left: At early-prometaphase, the centromere-spring is mechanically soft, sister centromere separation is low, and tension is also low. Center: At late-prometaphase, the mechanical stiffness of the centromere-spring is unchanged, but sister centromere separation increases substantially, which raises the median tension and narrows the range. Right: At metaphase, while sister centromere separation is similar to that in late-prometaphase, the stiffness of centromere-spring becomes displacement-dependent, thus amplifying both the magnitude and the range of the tension signal. The mean spring constant at each stage is taken from Fig. 2b. The median and IQR for sister centromere separation and centromere force were calculated from the distributions shown in Fig. 4c–f

To understand the downstream effects of centromere mechanical maturation, we characterized force transmission at the centromere during prometaphase and at metaphase. As described above, we defined the force transmission at the centromere as the tension or compression in the centromere-spring (*F*_C_; Fig. 5a), which may ultimately act as a mechanical signal to the cell, broadcasting the state of chromosome-spindle attachments (Fig. 1b)^4^. Tension and compression in a spring were calculated using “Hooke’s Law”, in which force (*F*_C_) is the product of the spring constant ($\bar \kappa$, as calculated above), and the displacement (*d*) (Fig. 5a, right; see Methods).

We began by evaluating the range of possible centromere force magnitudes at each mitotic stage, based on the range of displacement values that we observed in each case (Fig. 4d), and on our displacement-specific spring constant estimations for each mitotic stage (Fig. 4f–h; see Methods). We found that the dynamic range in tension at the centromere increased by 251% (Fig. 5b, shaded area) for a metaphase chromosome (Fig. 5b, solid line) as compared to a late-prometaphase chromosome (Fig. 5b, dotted line), from a total range in magnitudes of 67.6 pN at late-prometaphase to 237 pN at metaphase (Fig. 5b).

Thus, for an individual chromosome, the shift to a displacement-dependent spring at metaphase has a profound effect on the magnitude of change in the centromere-spring’s tension signal in response to even a small change in spring displacement. For example, when the centromere-spring of a late-prometaphase chromosome is stretched from 100 nm to 200 nm above its rest length, the magnitude of centromere tension increases by only ~10_;_pN, while the identical change in stretch for a metaphase chromosome increases the magnitude of tension by ~26 pN. This difference represents a 160% increase in tension for a metaphase chromosome as compared to a late-prometaphase chromosome for an identical centromere-spring displacement value. Moreover, as a result of the centromere-spring’s shift to a displacement-dependent spring during metaphase, the magnitude of centromere tension increases exponentially as the centromere-spring is stretched farther from its rest length (Fig. 5b).

### Centromere force distribution is shifted at metaphase

To assess the effect of centromere mechanical maturation on the overall observed distribution of centromere force values (*F*_C_) during mitotic progression, we compared chromosomes at early-prometaphase, late-prometaphase, and metaphase. As cells transitioned from early- to late-prometaphase, we observed a significant shift in the centromere force distribution (Fig. 5d), which mirrored the increase in sister centromere separation (Fig. 5c). In contrast, as cells transitioned from late-prometaphase to metaphase, the distribution of centromere force shifted significantly (Fig. 5f), despite no significant difference in sister centromere separation (Fig. 5e). Specifically, the median magnitude of centromere tension increased 120% from late-prometaphase to metaphase (KW_Stat_= 142.2, one-way, *p* = 9*e*^−33^). Further, consistent with our dynamic range estimates for individual chromosomes (Fig. 5b), the range of tension magnitudes for metaphase chromosomes increased approximately 304% as compared to the range for late-prometaphase chromosomes (Fig. 5f; BF_Stat_ =  32.5, Brown–Forsythe test, *p* = 2E-8).

Of note, our estimates of mitotic centromere tension are consistent with those reported in studies utilizing either physical or biochemical chromosome manipulations. For example, in R.B. Nicklas’ classic microneedle manipulation experiments, the stretching force on a prometaphase grasshopper chromosome was estimated at 50 pN^44^. More recently, a FRET-based molecular force sensor was used in mitotic drosophila S2 cells to estimate force at a single kinetochore at 135–680 pN^45^, and laser microsurgery experiments in human cells have yielded estimates of approximately 100–300 pN at a single kinetochore^15^. Additionally, our estimates are on the same order as estimates for centromere tension that were inferred from kinetochore tracking data in metaphase (mean tension = 6.9 pN)^46^.

Taken together, our analysis of the temporal dynamics in the mechanical properties of the centromere-spring, and the corresponding force transmission at the centromere, describe a complex process that we term “centromere mechanical maturation” (Fig. 5g). Our data describe the centromere-spring during early mitosis as mechanically soft and undergoing a fluid process of displacement; as a result, tension at the centromere is low, but also variable (Fig. 5g, left). As the cell progresses into late-prometaphase, the centromere-spring remains soft but is increasingly stretched, creating a greater and more uniform magnitude of tension (Fig. 5g, center). Finally, as the cell reaches metaphase, the centromere’s mechanical function matures fully: centromere-spring stiffness increases substantially in a displacement-dependent manner, the dynamic range of the centromere’s tension signal increases, and the magnitude of tension at the centromere is strongly amplified (Fig. 5g, right).

### Centromere mechanical maturation is disrupted in cancer cells

**Fig. 6:**
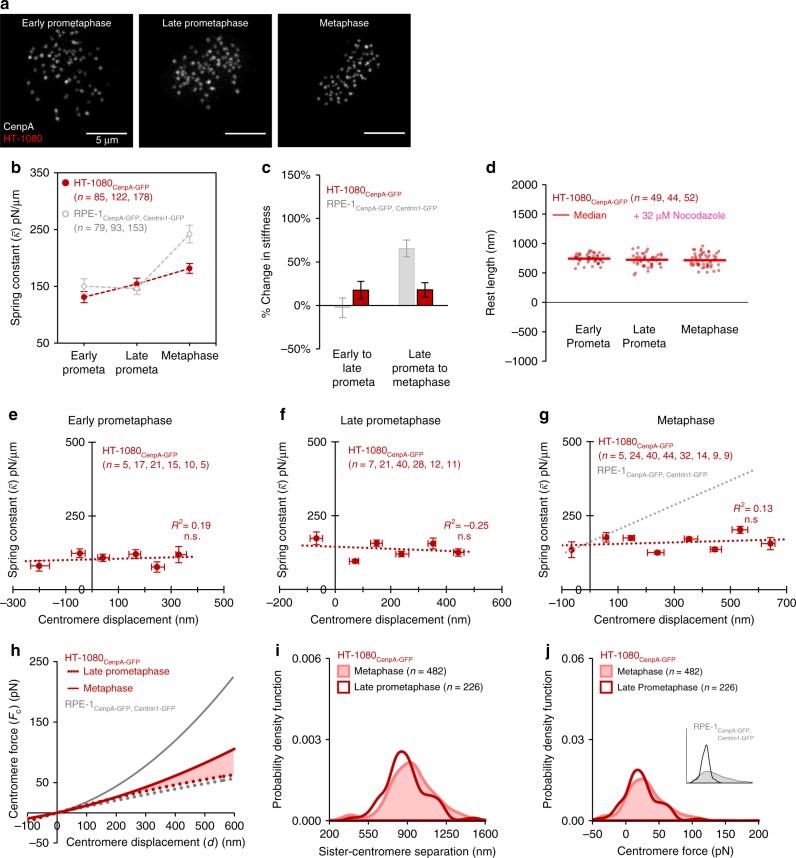
Mechanical maturation of the centromere is altered in cancer cells. **a** Live cell imaging of HT-1080 cells expressing CenpA-GFP at the outer centromere. Images are maximum intensity projections from full-cell-volume image series, and are representative of the described mitotic stages: early-prometaphase (left), late-prometaphase (center), and metaphase (right). Scale bar, 5 μm. **b**, **c** Mean spring constant by mitotic stage for HT-1080 cells (**b**, red) and the percent change in the mean stiffness of the centromere-spring across mitotic stages (**c**, red; all *n*-values as in panel **b**). Data from RPE-1 cells included for comparison (**b**, **c**; gray). Error bars represent standard error. **d** Quantification of the centromere-spring rest length by mitotic stage for HT-1080 cells. Data points reflect individual chromosome data points pooled across three independent experiments, the group median is shown in red. **e**–**g** Relationship between the displacement of the centromere-spring and its stiffness at early-prometaphase (**e**), late-prometaphase (**f**), and metaphase (**g**) for HT-1080 cells. Chromosomes were subgrouped by displacement in 100 nm intervals starting at the group minimum. Only subgroups with four or more chromosomes are shown. Each data point reflects the subgroup’s median displacement and mean spring constant; *X*-axis error bars represent IQR and *Y*-axis error bars represent standard error. The least-squares regression fit is indicated by the dotted line. Exact *p* values from linear regression fit are shown for models meeting statistical significance, all others are indicated as non-significant (n.s.). The least-squares regression fit line for RPE-1 chromosomes at metaphase is shown for comparison (**g**, dotted gray line). **h** The dynamic range in force transmission for a late-prometaphase chromosome (dotted red line) versus a metaphase chromosome (solid red line) for HT-1080 cells. The shaded region reflects the increase in dynamic range ( + 49.3%) between late-prometaphase and metaphase. The dynamic range for a RPE-1 chromosome at late-prometaphase (dotted line) and metaphase (solid line) are shown in gray for comparison. **i**, **j** Probability density functions for individual observations of **i** sister centromere separation (*s*) and **j** centromere force (*F*_C_) at late-prometaphase (dark red line) and metaphase (light red line, shaded area) for HT-1080 cells. Inset: RPE-1 centromere force distributions at late-prometaphase (black line) and metaphase (gray line, shaded area) are shown for comparison. All *n*-values listed are chromosome numbers

We next sought to examine whether centromere mechanical maturation could be disrupted in disease processes. Dysregulated cell division is pathognomonic of cancer, and a range of abnormal mechanical phenotypes, including altered microtubule dynamics, cellular viscosity, and spindle geometry, have been related to cancer^47–49^. Thus, we applied our protocol for characterizing centromere mechanics in a near-diploid, fibrosarcoma cell line (HT-1080) that proceeds through mitosis in stages broadly equivalent to the diploid, non-cancerous RPE-1 cells (Fig. 6a).

In HT-1080 cells, the stiffness of the centromere-spring increased by 18% from early- to late-prometaphase (131.1 ± 9.78 pN to 154.1 ± 10.4 pN/μm; Fig. 6b, c, red). At metaphase, centromere-spring stiffness increased an additional 18% to 181.7 ± 8.61 pN/μm. In comparison, recall that in RPE-1 cells, the centromere-spring stiffness changed by approximately −3% during prometaphase and by approximately 66% at metaphase (Fig. 6b, c, gray data). This difference suggested that centromere mechanical maturation may be disrupted in the HT-1080 cancer cells.

We next evaluated the relationship between the centromere-spring’s displacement and its stiffness, and found that during early- and late-prometaphase the centromere-spring was displacement-independent, consistent with RPE-1 cells (Fig. 6d–f). However, in stark contrast to the RPE-1 cells, in which the centromere-spring switched to a displacement-dependent spring at metaphase, in HT-1080 cancer cells the displacement-independent centromere-spring persisted into metaphase (Fig. 6g, red). These alterations to the centromere’s mechanical maturation significantly suppressed the centromere’s tension signal in the HT-1080 cells: the dynamic range of the tension signal for a metaphase HT-1080 chromosome was only 49% greater than that of a late-prometaphase chromosome, as compared to 251% increase for the RPE-1 cells (Fig. 6h, gray versus red). Consequently, the centromere force distribution for metaphase chromosomes in the HT-1080 cells was largely unchanged from late-prometaphase to metaphase (Fig. 6i, j), and lacked the increase in magnitude and range that was observed for the RPE-1 cells (Fig. 6j, inset).

To test whether these differences between cancer and non-cancer cells were specific to the fibrosarcoma HT-1080 cells, we repeated our experiments in three additional cancer types: adenocarcinoma (HeLa cell line, Supplementary Fig. 2), glioblastoma (U-87 cell line, Supplementary Fig. 3), and osteosarcoma (U2OS cell line, Supplementary Fig. 4). This panel of cell lines provides a broad representation of cancer tissue types that grow with comparable cell culture phenotypes. Strikingly, we found that the alterations in centromere mechanical maturation identified in the HT-1080 cells were broadly conserved in each cancer cell line, as centromere-spring stiffness tended to increase weakly at metaphase (Supplementary Figs. 2–4), and, in each cell line, the transition to a displacement-dependent centromere-spring at metaphase was absent (Supplementary Figs. 2–4). As a result, and in stark contrast to the RPE-1 cells, the centromere force distribution profiles in the cancer cell lines were largely indiscriminate between late-prometaphase and metaphase (Supplementary Figs. 2–4).

**Fig. 7:**
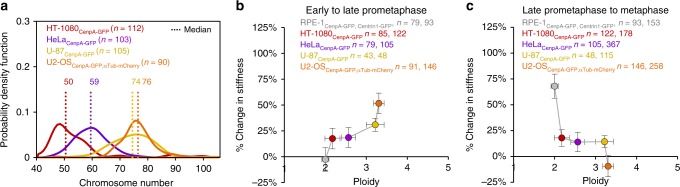
Mechanical maturation of the centromere correlates with ploidy in cancer cells. **a** Probability density functions for individual observations of total chromosome number in HT-1080, HeLa, U-87, and U2OS cells. Sample sizes (*n*) reflect the number of cells per sample. Dotted lines and data labels indicate the median chromosome number in each cell line. **b**, **c** Relationship between percent change in mean centromere stiffness and ploidy for early- to late-prometaphase (L) and late-prometaphase to metaphase (M) across the four cancer cell lines, with data from non-cancerous RPE-1 cells shown for comparison. *X*-axis error bars represent IQR; *Y*-axis error bars represent standard error. All n-values listed are chromosome numbers. See also Supplementary Figs. 2–4 and 6–7

Intriguingly, when we evaluated the median chromosome number for each cancer cell line (Fig. 7a), our results hinted at a potential trend in centromere-spring stiffness maturation with increasing chromosome number. Here, while the percent change in centromere-spring stiffness from early to late-prometaphase tended to increase with increasing chromosome number (Fig. 7b), the percent change in centromere-spring stiffness from late-prometaphase to metaphase tended to decrease with increasing chromosome number (Fig. 7c). Since an important outcome of centromere mechanical maturation was an increase in both the magnitude and the range of tension at metaphase, this result suggested that increased chromosome numbers could potentially disrupt force transmission at metaphase.

### Aneuploidy impairs maturation of centromere-spring stiffness

**Fig. 8:**
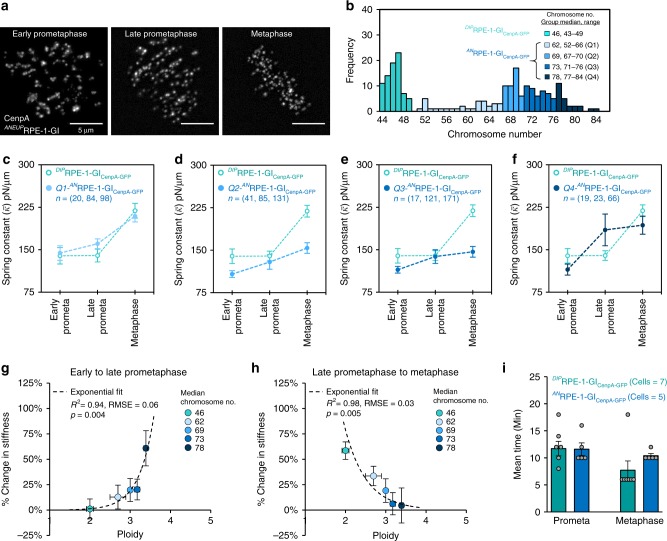
Aneuploidy impairs centromere mechanical maturation. **a** Live cell imaging of ^*AN*^RPE-1-GI cells expressing CenpA-GFP at the outer centromere. Images are maximum intensity projections from full-cell-volume image series, and are representative of the described mitotic stages: early-prometaphase (left), late-prometaphase (center), and metaphase (right). **b** Histogram of chromosome numbers for a sample of RPE-1-GI cells differentiating between diploid cells and aneuploid cells. ^*AN*^RPE-1-GI cells are also subgrouped by quartile (Q1, light blue–Q4, dark blue$;n_{{\mathrm{eP}}}^{{\mathrm{Ce}}}$ = 48, $n_{{\mathrm{lP}}}^{{\mathrm{Ce}}}$ = 80, $n_M^{{\mathrm{Ce}}}$ = 110). **c**–**f** Mean spring constant by mitotic stage for ^*AN*^RPE-1-GI chromosomes by quartile of increasing chromosome numbers as in panel **b** (Q1, light blue filled data point–Q4, dark blue filled data point). Data from ^*DIP*^RPE-1-GI cells included for comparison (green unfilled data points; *n* = 131 EP, 136 LP, 178 M, all panels). Error bars represent standard error. All *n*-values listed are chromosome numbers. **g**, **h** Relationship between percent change in mean stiffness from **g** early to late-prometaphase or **h** late-prometaphase to metaphase and cell ploidy for RPE-1-GI cells. Data points include ^*DIP*^RPE-1-GI cells (green) and ^*AN*^RPE-1-GI cells by quartile (Q1, light blue–Q4, dark blue). *X*-axis error bars represent IQR; *Y*-axis error bars represent standard error. Fit to an exponential regression model is indicated by the dashed-line and the listed *R*^2^, *RMSE*, and *p*-values. **i** Comparison of mean mitotic timing between ^*DIP*^RPE-1-GI cells (teal) and ^*AN*^RPE-1-GI cells (blue). Sample sizes (*n*) reflect the number of cells in each group. Error bars represent standard error; dots represent individual cells. See also Supplementary Figs. 5 and 7

To more directly test whether aneuploidy impairs mechanical maturation of the centromere, we repeated our experiments in a non-cancerous cell line containing a diversity of ploidy states. We obtained an otherwise untransformed RPE-1 cell line that contains populations of both diploid and aneuploid cells resulting from an unprovoked genome instability event^50^, and transformed it to express CenpA-GFP (RPE-1-GI, Fig. 8a). We applied our protocol for evaluating centromere mechanics to a large sample of cells and then during analysis, we subdivided cells as either diploid or aneuploid based on their specific chromosome number (Fig. 8b).

While the signature hallmarks of centromere mechanical maturation were present in the diploid RPE-1-GI cells (Fig. 8c, green data points), consistent with our RPE-1 cell line, the pattern of centromere-spring stiffness maturation over mitosis was increasingly altered at higher chromosome numbers in the aneuploid cells (Fig. 8c–h, blue data points). Of note, among the cells with the highest chromosome numbers (median = 78, range = 77–84), the pattern of stiffness change diverged significantly (Fig. 8f, dark blue data points). Interestingly, this pattern was consistent with the results obtained from the cancer cell line with the highest chromosome number (U2OS cell line, median = 76; Supplementary Fig. 4B). However, most striking observation was the near exponential disruption in the magnitude of centromere-spring stiffness maturation from late-prometaphase to metaphase as a function of increasing chromosome number (*R*^2^ = 0.98, nonlinear regression, *p* = 0.005, Fig. 8h).

Importantly, we found that mitotic timing between aneuploid and diploid RPE-1-GI cells did not contribute to the differences we observed in their centromere maturation patterns (Fig. 8i, mean = 22.0 min, SEM = 0.9 min), as compared to diploid cells (Fig. 8i, mean = 19.4 min, SEM = 1.6 min). Further, we used a small-molecule inhibitor specific to Aurora-B activity (AZD-1152)^51^ to test whether inhibition of the kinase’s activity in diploid cells resulted in a centromere mechanical maturation pattern similar to that which we observed in the aneuploid cells, and found that there was no effect of AZD-1152 treatment on the baseline centromere stiffness at late-prometaphase (*z* = 0.09, *z*-test, *p* = 0.94), and that suppressing Aurora-B kinase activity did not affect centromere maturation between late-prometaphase and metaphase (Supplementary Fig. 5A–C; *z* = 0.4, *z*-test, *p* = 0.70).

Finally, to understand whether the relationship between aneuploidy and centromere mechanical maturation was conserved in a non-human mammalian species, we re-examined the muntjac cell line. This line contains a minority subpopulation of tetraploid cells (Supplementary Fig. 5D), which resulted from the cell line’s immortalization with SV40 (ref. ^52^). When we reapplied our protocol for evaluating centromere mechanics in this cell line, selecting for the tetraploid cells (Supplementary Fig. 5E), we found that centromere mechanical maturation was also impaired in the aneuploid cells. Here, centromere stiffness increased 6% between late-prometaphase and metaphase in the tetraploid cells, as compared to the 72% increase observed for the diploid cells (Supplementary Fig. 5F, G). We conclude that aneuploidy impairs maturation of the centromere-spring’s stiffness during mitosis in a dose-dependent manner.

### Disrupted centromere mechanical maturation dampens tension in aneuploid cells

**Fig. 9:**
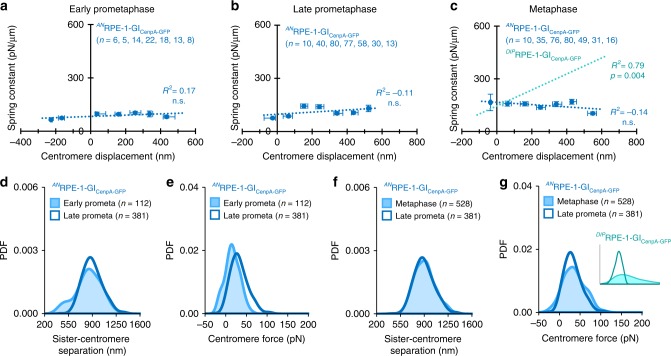
Disrupted centromere mechanical maturation dampens metaphase tension. **a**–**c** Relationship between the displacement of the centromere-spring and its spring constant at early-prometaphase (**a**), late-prometaphase (**b**), and metaphase (**c**) for aneuploid RPE-1-GI cells. Chromosomes were subgrouped by displacement in 100 nm intervals starting at the group minimum. Only subgroups with four or more chromosomes are shown. Each data point reflects the subgroup’s median displacement and mean spring constant; *X*-axis error bars represent IQR; *Y*-axis error bars represent standard error. The least-squares regression fit is indicated by the dotted line and the listed *R*^2^ value. Exact *p* values from linear regression fit are shown for models meeting statistical significance; all others are indicated as non-significant (n.s.). The least-squares regression fit line for diploid RPE-1-GI chromosomes at metaphase is shown for comparison (**c**, dotted green line). **d**, **e** Probability density functions for individual observations of **d** sister centromere separation and **e** centromere force at early-prometaphase (light gray line, shaded area) and late-prometaphase (black line) for aneuploid RPE-1-GI cells (^*AN*^RPE-1-GI). **f**, **g** Probability density functions for individual observations of **f** sister centromere separation and **g** centromere force at late-prometaphase (dark blue line) and metaphase (light blue line, shaded area) for aneuploid RPE-GI cells. Inset: Centromere force distributions at late-prometaphase (dark teal line) and metaphase (light teal line, shaded area) from diploid RPE-1-GI cells (^*DIP*^RPE-1-GI) are shown for comparison. See also Supplementary Figs. 5 and 7. All *n*-values listed are chromosome numbers

We next assessed whether impairment of the centromere-spring’s stiffness maturation in the aneuploid RPE-1 cells also affected the magnitude and dynamic range of metaphase tension. As we observed in the cancer cells, the metaphase transition to a displacement-dependent spring was absent in the aneuploid RPE-1-GI cells (Fig. 9a–c), and thus the magnitude and range of centromere tension at metaphase was significantly dampened (Fig. 9d–g). Importantly, amplification of centromere tension at metaphase remained intact in the diploid RPE-1-GI cells (Fig. 9g, inset). Thus, our results demonstrate that aneuploidy directly impacts the magnitude and range of metaphase tension through a disruption in the centromere’s mechanical maturation process.

### Disrupted centromere mechanical maturation correlates with merotelic chromosomes in anaphase

**Fig. 10:**
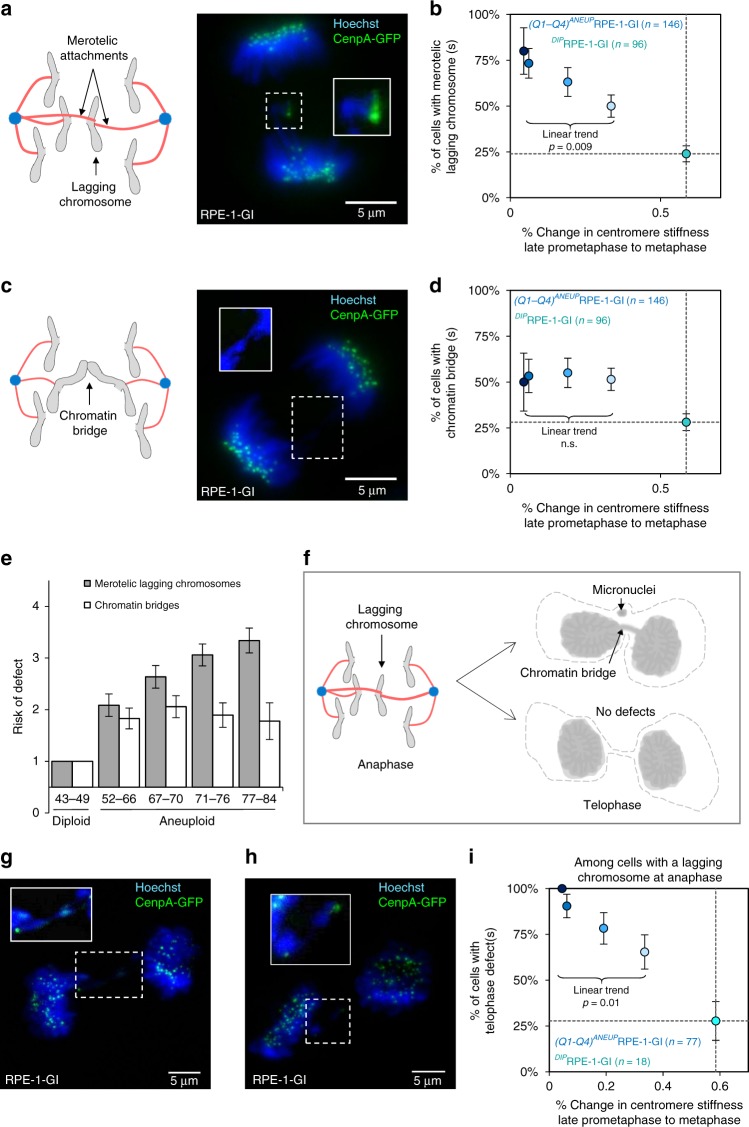
Impaired centromere mechanical maturation alters mitotic outcomes. **a** Graphical representation (left) of a lagging chromosome at anaphase. Representative image (right) of an anaphase lagging chromosome (outlined region) from live cell imaging of an ^*AN*^RPE-1-GI cell expressing CenpA-GFP at the outer centromere and stained with Hoechst. **b** Relationship between percent change in mean centromere stiffness from late-prometaphase to metaphase and percent of cells with lagging chromosomes at anaphase across ^*DIP*^RPE-1-GI cells (green) and ^*AN*^RPE-1-GI cells by quartile (CMH_Stat_ = 6.8, Cochran–Mantel–Haenszel test, *p* = 0.009). **c** Graphical representation (left) of a chromatin bridge at anaphase. Representative image (right) of an anaphase chromatin bridge (outlined region) from live cell imaging of an ^*AN*^RPE-1-GI cell expressing CenpA-GFP at the outer centromere and stained with Hoechst. **d** Relationship between percent change in mean centromere stiffness from late-prometaphase to metaphase and percent of cells with chromatin bridges at anaphase across ^*DIP*^RPE-1-GI cells (green) and ^*AN*^RPE-1-GI cells by quartile (CMH_Stat_ = 0.01, Cochran–Mantel–Haenszel test, *p* = 0.9). **e** Comparison of relative risk ratios for tension-based attachment defects (lagging chromosomes) versus tension-independent nondisjunction defects (chromatin bridges) at anaphase across ^*AN*^RPE-1-GI cells by quartile, normalized to ^*DIP*^RPE-1-GI cells. **f** Graphical representation of experimental scheme for generating data shown in **i**. Cells with lagging chromosomes at anaphase were followed to telophase and assed for the presence of persistent defects at telophase including micronuclei and chromatin bridges. **g** Image of a telophase chromatin bridge (outlined region) from live cell imaging of a Hoechst-stained ^*AN*^RPE-1-GI cell expressing CenpA-GFP at the outer centromere. **h** Image of micronuclei at telophase (outlined region) from live cell imaging of a Hoechst-stained ^*AN*^RPE-1-GI cell expressing CenpA-GFP at the outer centromere. **i** Relationship between percent change in mean centromere stiffness from late-prometaphase to metaphase and percent of cells with telophase defects across ^*DIP*^RPE-1-GI cells (green) and ^*AN*^RPE-1-GI cells by quartile; CMH_Stat_ = 6.5, Cochran–Mantel–Haenszel test, *p* = 0.01. Analysis included only those cells with a lagging chromosome at anaphase. The scale bar for all images is 5 μm. All error bars represent standard error. All *n*-values listed are number of cells. Exact *p* values are shown for models meeting statistical significance; all others are indicated as non-significant (n.s.)

Finally, to assess the functional significance of centromere mechanical maturation and its effect on tension signaling during mitosis, we analyzed the frequency of mitotic defects at anaphase and telophase in cells with impaired centromere mechanics. To control for the possibility that aneuploidy alone might increase the frequency of mitotic defects, we compared tension-based kinetochore-microtubule attachment defects with tension-independent cohesion-based defects. Specifically, chromosome attachment defects that arise from impaired tension-dependent error detection and correction processes were assessed by counting the number of anaphase cells with lagging chromosomes^53^ (Fig. 10a), which result from aberrant, merotelic kinetochore-microtubule attachments at metaphase^54^. It has been previously shown that tension signaling is critical to resolving and properly separating merotelically attached chromosomes^55^. Tension-independent defects were assessed by counting anaphase cells with chromatin bridges (Fig. 10c), which result from persistent cohesion between sister-chromatid arms during anaphase.

Among cells with proper centromere mechanical maturation, the rates of lagging chromosomes and chromatin bridges at anaphase were similar (24 versus 28%; Fig. 10b, d, green data points). Strikingly, the rate of lagging chromosomes increased significantly with centromere mechanical maturation impairment (CMH_Stat_ = 6.8, Cochran–Mantel–Haenszel test, *p* = 0.009), from 50% among cells with a mild impairment phenotype up to 80% among those with the most severe impairment (Fig. 10b, blue data points). In comparison, the frequency of chromatin bridges was constant across the range of centromere mechanical maturation impairment phenotypes (50–55%; Fig. 10d, blue data points; CMH_Stat_ = 0.01, Cochran–Mantel–Haenszel test, *p* = 0.9). Thus, at anaphase, only defects associated with impaired tension signaling increased significantly across aneuploid cells with increasingly severe centromere mechanical maturation impairment phenotypes (Fig. 10e).

As lagging chromosomes will often resolve during anaphase, with sister chromatids ultimately segregating to the correct daughter cell^56^, we followed cells with lagging chromosomes at anaphase into telophase to look for further evidence of chromosome missegregation (Fig. 10f). We found that the frequency of persistent errors at telophase also increased significantly with centromere mechanical maturation impairment (Fig. 10g–i; CMH_Stat_ = 6.5, Cochran–Mantel–Haenszel test, *p* = 0.01). Thus, our results suggest that centromere mechanical maturation may impact overall genome stability by increasing the risk of tension-based attachment errors such as merotelic lagging chromosomes at anaphase and resulting segregation defects at telophase.

## Discussion

In this work, we found that the centromere stiffness increases during mitotic progression with signature pattern that is conserved across human and non-human cell lines. This is consistent with findings by Jaqaman et al.^10^, who showed that a change in the mechanical linkage between sister chromatids during mitotic progression could explain the reduction in chromosome oscillation speed that was observed at metaphase^10^. Overall, our results speak to a highly nuanced view of the centromere’s mechanical function, and they suggest that the centromere is a sophisticated contributor to tension signaling in mitosis. Specifically, we found that at metaphase, when tension may play a critical role in preventing chromosome segregation errors in anaphase^4^, mechanical maturation of the centromere-spring augments both the magnitude and the dynamic range of centromere tension.

Among cells with intact centromere mechanical maturation, we found that even small changes in displacement of the centromere-spring at metaphase led to large changes in the magnitude of tension present at the centromere (Fig. 5b). This type of tension-based signal amplification would allow the cell to distinguish subtle differences in the configuration of microtubule attachments at the kinetochore, such as in the case of merotelic chromosomes, which may have only a few incorrect attachments. Conversely, in cells where the centromere failed to mature mechanically, we observed a lower magnitude of tension, and, importantly, the dynamic range of the centromere’s tension signal was dampened. Importantly, these cells were at increased risk for tension-based kinetochore-microtubule attachment defects, and these defects increasingly persisted into telophase as chromosome segregation defects (Fig. 10). This elevated rate of attachment errors and segregation defects suggests that when centromere mechanical maturation is disrupted, the cell’s ability to detect small changes in microtubule attachments at the kinetochore, and to selectively destabilize inaccurate attachments, is reduced. This is consistent with reports that kinetochore-microtubule attachments are hyper-stabilized in cancer cells, leading to reduced error-correction processes, and eventually producing segregation defects^43,47^. Thus, centromere mechanical maturation may impact overall genome stability through chromosome segregation outcomes that rely on tension signaling.

Importantly, the temporal dynamics of centromere mechanical maturation suggest that observations of sister centromere separation (*s*, Fig. 4a) can be decoupled from the actual magnitude of tension present at the centromere (*F*_C_; Fig. 4A). For example, we found that for RPE-1 cells, a metaphase chromosome with a sister centromere separation of 1000 nm exhibited a magnitude of tension that was approximately three times higher than a similarly stretched late-prometaphase chromosome. This decoupling indicates that caution should be used in assigning sister centromere separation as a proxy measurement for tension in mammalian cells. Cell line-specific differences in centromere mechanical maturation further confound the relationship between sister centromere separation and tension in mammalian cells. For example, we found that tension for a metaphase HeLa chromosome with a sister centromere separation of 1000 nm was less than 65% of that of a metaphase RPE-1 chromosome. These dichotomies suggest a paradigm shift in how centromere tension is conceptualized and defined.

Our findings also suggest that further exploration into the effects of aneuploidy on chromosomes’ mechanical function during mitosis may be fruitful. Aneuploidy is a hallmark of cancer, and is an emerging target for chemotherapeutic agents^57^. While the effects of aneuploidy on gene expression and regulation have been extensively studied, our findings open the door to new avenues of inquiry into its broader effects on chromosome biology. Moreover, while the causal relationship between aneuploidy and carcinogenesis is not fully understood, our results suggest a role for centromere mechanics in amplifying chromosomal instability in aneuploid cells. Interestingly, recent findings suggest that increasing the rate of chromosome missegregation could be exploited as a chemotherapeutic target, as inducing high rates of missegregation have been shown to suppress cancer progression^58^. In our work with budding yeast, we found that Cisplatin, a drug which crosslinks DNA, altered normal centromere stiffness^59^. As Cisplatin is used clinically to treat a variety of cancer types, this raises interesting questions regarding the role of centromere mechanics in altering chromosome segregation outcomes as mechanism for inhibiting cancer progression.

Past studies have identified the structural maintenance of chromosomes (SMC) proteins, condensin and cohesin; DNA decatenation via Topoisomerase II-alpha; and higher-order organizing of centromeric chromatin as factors that influence the elasticity and tensile strength of the centromere^26,60–62^. In particular, depletion of condensin II and cohesin at the centromere has been shown to alter normal centromere structure, leading to increased numbers of lagging chromosomes^63,64^, similar to what we observed here. Thus, a parsimonious explanation for the apparent dose-dependent relationship between aneuploidy and impairment of centromere mechanical maturation may be a dilution of these factors relative to the increase in chromosome number. In essence, protein availability (e.g. SMC proteins or chromatin remodeling factors) may become limiting in the chromosomes’ ability to undergo the structural changes necessary to support their mechanical function. For example, both experimental and computational findings have identified condensin, cohesin, and topoisomerase II as key mediators of the progressive chromosome condensation that occurs during mitotic progression and culminates with global compaction at metaphase^33^. Results from polymer simulations of chromosome dynamics have indicated that chromatin loop extrusion could mediate this process^65^. Correspondingly, recent work using mathematical modeling of the budding yeast mitotic spindle has proposed that pericentromeric chromatin loops cross-linked with cohesin and condensin could act as a nonlinear spring in mitotic force balance, and that specific chromatin configurations may actively stiffen the centromere during mitotic progression^62,66,67^. Thus, it is possible that increased chromosome condensation mediated by chromatin looping at metaphase could enable the maturation of the centromere to a nonlinear spring. Further, microtubule-mediated springs within the mitotic spindle^68,69^ could potentially contribute to our observations of centromere-spring stiffening in metaphase. We note that our spindle constraint stiffness data, gathered from measurements of non-sister centromere fluctuations, suggests that microtubule-based spindle constraints themselves do not substantially contribute to our centromere maturation results (Supplementary Fig. 1A). However, further experiments to more directly measure spindle-based stiffness constraints will be important for evaluating a potential contribution of microtubule‐mediated springs to our centromere-spring stiffness maturation results. Dissecting the contributions of these molecular actors and structural processes to the mechanical function of the centromere-spring, as well as determining whether altered protein expression can restore the centromere’s mechanical maturation in impaired cells, will require careful and detailed study, and is an important next step.

In summary, our work characterizes the mechanical maturation of the centromere in mammalian cells and its effect on centromere tension. Our results indicate that a change in the centromere’s mechanics occurs independently of spindle dynamics during mitotic progression, as centromere stiffness did not change with the spatial positioning of the chromosome, the state of kinetochore-microtubule attachments, the activity of Aurora-B kinase, or the presence of microtubule dynamics. Our findings also describe how these processes differ in cancer and aneuploidy, and the consequential role that centromere mechanics may play in overall genome stability through impacting chromosome segregation outcomes.

## Methods

### Cell lines

Cells were grown in Dulbecco’s minimum essential medium (Thermo Fisher Scientific, Waltham, MA) with 10% fetal bovine serum and 1% penicillin and streptomycin. Cultures were grown at 37 °C in 5% CO_2_ in a humidified incubator, and maintained at low passage numbers. HeLa-k cells were maintained in 0.5 µg/ml puromycin (Thermo Fisher Scientific, Waltham, MA). U-87 cells were maintained in 0.4 µg/ml G148 (Thermo Fisher Scientific, Waltham, MA). To generate cell lines with fluorescent centromeres, recipient cells were transduced with a CenpA-GFP lentivirus. This virus was generated in HEK293T cells transfected with a CenpA-GFP-LentiLox 3.1 plasmid (a gift from Dr. Alexey Khodjakov). Flow cytometry was used to isolate cells with a GFP fluorescent signal, and clonal dilution was used to isolate populations with a consistent centromere signal across all cells. RPE-1_CenpA-GFP,Centrin-1_ (ref. ^35^), HeLa-K_CenpA-GFP_ (ref. ^10^), U2OS_CenpA-GFP,α-tubulin-mcherry_ (ref. ^70^), U-87_CenpA-GFP_, RPE-1-GI^50^, and RPE-1^CenpA−/+GFP^ (ref. ^38^) cell lines were gifts of Drs. Alexey Kohdjakov, Andrew McAinsh, Helder Maiato, Eric Van Dyck, Bo Huang, and Lars Jansen, respectively. HT-1080_CenpA-GFP_^71^ and SVM_CenpA-GFP,Centrin-1-GFP_ cell lines were gifts from Dr. Duncan Clarke. HeLa cells are listed in the database of commonly misidentified cell lines, but we included this cell line in our analysis of other cancer cell lines because of its wide usage in mitosis research. While this cell line was not authenticated or tested, the chromosome number was established for all HeLa cells used.

### Live cell imaging

Cells were cultured to sub-confluence on glass-bottom dishes (MatTek, Ashland, MA) and then transferred to CO_2_-independent medium (Thermo Fisher Scientific, Waltham, MA) supplemented with GlutaMAX (Thermo Fisher Scientific, Waltham, MA), 10% FBS, and 1% penicillin/streptomycin. Cells were imaged on a Nikon Eclipse Ti inverted microscope with a motorized X–Y stage with a Piezo insert for *z*-plane movement, using a 488 nm laser line, and a Nikon CFI Plan Fluor ×20 0.5 NA air objective and a CFI Apochromat ×100 1.49 NA oil objective. The laser angle was adjusted to a customizable depth to increase penetration of the evanescent field into the sample, while optimizing the signal to noise ratio. An EMCCD camera (iXon3, Andor Technologies) with a 30 MHz pixel readout speed fitted with a ×2.5 projection lens was used to capture images with an effective pixel size of 64 nm^2^. Samples were maintained at 37 °C using a heated objective and stage controlled by an Okolab H401-T-DUAL-BL temperature controller (OKOLAB USA Inc., Burlingame, CA), and monitored with a fine gauge thermocouple imbedded in the culture media. NIS Elements software was used for microscope control and image acquisition. Cultures were discarded at the end of each imaging session and were not reused for subsequent experiments.

### Centromere motion tracking protocol

To track centromere motion, we adapted a method previously described by our laboratory for use in budding yeast to the conditions present in mammalian cells. For a detailed discussion of the theory underlying our approach and the supporting rationale, see ref. ^26^. hTERT-RPE-1 cells, which are commonly used to represent a normal mitotic phenotype, were used for all assay development and validation steps. The outer centromere on each chromatid was visualized through stable expression of a centromere protein A (CenpA) GFP fusion protein. CenpA, a Histone 3 variant specific to the centromere, is loaded during G1 of the cell cycle, and does not otherwise turnover during the cell cycle^5,72^. It creates a constant and near diffraction-limited fluorescent centromere signal throughout mitosis which can be used to track motion of a pair of sister centromeres^12,73^. Mitotic cells were first identified using bright-field illumination at ×20 magnification. A full-cell-volume image series with a *z*-step of 250–300 nm and exposure time of 100–200 ms was taken for later review and determination of the cell’s mitotic stage. During analysis, metaphase cells that demonstrated a synchronized separation of sister centromere pairs during image acquisition were removed in order to exclude the effects of anaphase onset. Individual centromere pairs were then imaged using continuous single color acquisition on a single focal plane with a total magnification of ×250. The total acquisition duration for each image series was limited to 1–2 s to minimize sample photobleaching and signal to noise variability. A reduced region of interest (ROI) with an area of ~72 μm^2^ was used to achieve a frame rate of 150 Hz, for an effective exposure time of 0.0067 s. This frame rate provides a temporal resolution that exceeds the timescale of active forces, such as from molecular motors and microtubule dynamics, that act on the chromosome. For example, human chromosomes have been observed moving at velocities up to 300 nm/s during chromosome congression, and individual kinetochores have been reported to move at an average speed of 23 nm/s during chromosome oscillations and breathing^12,35^.

### Nocodazole treatment

Live cell imaging was used to identify cells at early-prometaphase, late-prometaphase and metaphase. Nocodazole (Thermo Fisher Scientific, Waltham, MA) was added to the culture dish to a final concentration of 32 μM, and dishes were then incubated on the microscope stage for 20 min. After incubation, the temperature was reduced to 25 °C to reduce thermal drift of unattached chromosomes, and full-cell-volume image series with a 250–300 nm *z*-step and a 100–200 ms exposure time were immediately acquired for the previously staged cells.

### Mitotic defects

Live cell imaging was used to identify cells at metaphase. A full-cell-volume image series with a *z*-step of 250–300 nm and exposure time of 50–200 ms was taken for later review and determination of the cell’s chromosome number. Cells were then followed into anaphase and telophase, with full-cell-volume images series taken for later review and assessment of visible defects.

### Western blots

RPE-1 cells were lysed using cynase-compatible lysis buffer with a final composition of 1% Triton X-100, 25 mM NaCl, 50 mM Tris pH 8.0, 10 mM MnCl_2_, and 50 U/ml cynase endonuclease (RiboSolutions, Inc., Cedar Creek, TX). Cell lysates were analyzed for protein concentration using a Nanodrop ND-1000 Spectrophotometer and the Pierce 660 Protein Assay Kit (Thermo Fisher Scientific, Waltham, MA). Aliquots were diluted in BRB80 and PAGE buffer. Samples were run on 15% SDS-PAGE gels and then transferred to PVDF membranes. Membranes were probed with primary antibodies for anti-CenpA (Cell Signaling Technology, Danvers, MA; catalog # 2186S; dilution 1:10,000), and anti-H4 (a gift from Dr. Judith Berman; anti-Histone H4 antibody dilution 1:5000), followed by a horseradish peroxidase-linked anti-rabbit secondary (Santa Cruz Biotechnology, Dallas, TX; catalog # SC2004) and then developed using chemiluminescence. For quantification, total CenpA intensity was normalized to total H4 intensity for each sample. See source data file for uncropped scans of the western blots.

### Software packages

ImageJ (Research Services Branch, National Institute of Mental Health, Bethesda, Maryland, USA) was used for image review and presentation and for western blot quantification. Image quantification was done in MATLAB (Mathworks, Natick, MA) using custom-written scripts (available upon request). Statistical analyses were performed using built-in MATLAB functions and with SAS 9.4 (SAS Institute, Cary, NC).

### Image processing

Raw, single focal plane images were processed with course and fine grain Gaussian filters for background subtraction and noise correction. For representative images of mitotic stages and treatment conditions, a maximum intensity projection was created in ImageJ using image stacks taken from the full-cell-volume image series.

### Chromosome counting

Full-cell-volume image series taken with a *z*-step of 250–300 nm and exposure time of 100–200 ms for cells at late-prometaphase or metaphase were reviewed using a custom MATLAB script. Imaging planes were averaged in the *z* direction with a bin size of two *z*-slices for a final voxel resolution of 64 nm×64 nm ×  500–600 nm, and then displayed sequentially as the user manually selected individual centromere centroids. Selected centroids from the three prior focal planes were projected over each image to prevent recounting a centromere visible in multiple focal planes. The total number of centromeres in each cell was divided by two to yield the cell’s chromosome number. Ploidy for a sample of cells from a specific cell line was calculated by dividing the group’s median chromosome number by the organism’s haploid number. Summary data for each cell line is displayed either as a histogram or a probability density function.

### Sister centromere rest length

Image series obtained after nocodazole treatment (see “Methods details” section) were reviewed using ImageJ to identify sister centromere pairs located in the same focal plane. 2D Gaussian mixture model fitting was then applied to cropped images of sister centromere pairs in order to identify the centroid of each CenpA spot, and the Euclidean distance between the two was calculated. For each cell line, data was collected across ≥3 independent experiments and then pooled by mitotic stage. The stage-specific rest length ($\overline {l_R}$) used during subsequent analyses was the mean distance obtained for all measured chromosomes at a specific stage.

### Sister centromere separation and centromere-spring displacement

Sister centromere separation was defined as the Euclidean distance between CenpA centroids of sister centromeres on chromosomes proceeding through mitosis unperturbed. Separation distances for individual chromosomes with sister centromeres located on the same focal plane were calculated using images series taken using continuous single color acquisition with a frame rate of 150 frames per second. The mean distance between centroids over the first 30 consecutive frames (200 ms total duration) was used to estimate the chromosome’s sister centromere separation (*s*) at that time point. This timeframe was chosen to minimize the effects of active motion in influencing the sister centromere separation values (Eq. (3)).

Centromere-spring displacement for an individual chromosome (*d*) was calculated by

1 $$d = s - \overline {l_{R}},$$

where $\overline {l_{R}}$ is the cell line’s stage-specific mean rest length and *s* is the measured separation between the sister centromere CenpA centroids, as described above.

### MSD analysis

To quantify the MSD of a CenpA-GFP tag on the outer centromere of a mitotic chromosome, we applied a method developed previously in our laboratory using budding yeast^26^. For a detailed discussion of the theory underlying this approach and the supporting rationale, see ref. ^26^. A similar approach has also been reported for quantifying the MSD of telomeres on human chromosomes at longer time frames (1–5 s) during interphase, and also applied to fluorescently-tagged gene loci on the chromosome arm^50^.

Image series obtained using the centromere motion tracking protocol (see “Methods details” section) were reviewed in ImageJ prior to inclusion in MSD analyses. As our rapid image acquisition rate precluded imaging in multiple focal planes (*z*-planes), we restricted our analyses to image series where both sister centromere pairs remained in focus on the focal plane throughout the image acquisition duration. To control for variation in measurement error across imaging sessions, we also restricted analyses to image series with MSD intercept values less than half a pixel (32 nm^2^). Since the MSD intercept is representative of measurement noise due to low signal intensity or other factors, this threshold ensured that measurement noise was minimized in our analysis. For the image series that met our inclusion criteria, the centroids for both CenpA-GFP spots within a sister centromere pair were localized in each frame with sub-pixel resolution using 2D Gaussian mixture model fitting. The Euclidean distance between the two centroids was then calculated and divided by $\surd 2$ to represent the motion of a single centromere. Thus, the spring constant estimate we ultimately derived for the centromere-spring represents an integration of the stiffness between the outer centromere regions of two sister centromeres. The MSD at each time interval from 0.0067 s up to 1 s was calculated as follows^25^:

2 $${\mathrm{{MSD}}}\left( t \right) = \frac{1}{n}\mathop {\sum }\limits_{j = 1}^n \left[ {\mathop {\sum }\limits_{i = j}^{j + \left( {t/t_{{\mathrm{{step}}}}} \right) - 1} R_i} \right]^2.$$

Here, *t* is the time interval, *t*_step_ is the time step size between two frames (0.0067 s under our imaging conditions), and *n* is the number of displacement measurements obtained from the image series.

To determine the average MSD for a sample of cells from each cell line at each mitotic stage, the inter-frame displacements were pooled for all chromosomes at each time interval from one (0.0067 s) to 150 (1 s).

### MSD curve fitting

To fit our MSD data to equations for different motion types, we used an approach previously described for fitting MSD data from a fluorescent tag at the telomeres of human chromosomes, as well as at specific chromosome gene loci^50^. In this approach, MSD data were consistent with an MSD mixed-motion model that included terms for confined diffusion, macroscopic diffusion, and active transport. Thus, our pooled MSD curves for time intervals up to 1 s were fitted by nonlinear least-squares regression to a mixed-motion MSD model^74^:

3 $${\mathrm{{MSD}}}\left( t \right) = \left\langle {\sigma ^2} \right\rangle \left( {1 - {\mathrm{e}}^{ - \tau /{\hat{\mathrm{o}}}}} \right) + {4D_{\mathrm{{macro}}}}{\it{t}} + v^2t^2 + b_\theta,$$

where *σ*^2^ is the net MSD, $\tau /{\hat{\mathrm{o}}}$ is a constant from which the microscopic diffusion coefficient can be derived, *D*_macro_ is the macroscopic diffusion coefficient, *v* is the velocity of active transport. An intercept ($b_\theta$) was also included and set to the experimental value for MSD($t = 0.0067\;{\mathrm{s}}$) as an estimate of the measurement error inherent in the assay^26^. The curves were fit with a constraint for positive parameters for *σ*^2^, $\tau /{\hat{\mathrm{o}}}$, *v*, and $D_{{\mathrm{{Macro}}}}$. The fit results for pooled MSD curves by mitotic stage for each cell line are presented in Supplemental Figs. 7–10. As *R*^*2*^ values can provide a less reliable measure of goodness of fit for nonlinear regression models, both the *R*^*2*^ and root mean squared error (RMSE) values are included.

For short timescale MSD results (*t* < 0.2 s), the macroscopic diffusion and the active transport terms in Eq. (3) (both of which depend on *t*) become negligible. Thus, for MSD curves from individual chromosomes, as well as pooled MSD curves, the initial 0.1–0.2 s of the curve were also fitted by nonlinear least-squares regression to a model for confined diffusion:

4 $${\mathrm{{MSD}}}\left( t \right) = \left\langle {\sigma ^2} \right\rangle \left( {1 - {\mathrm{e}}^{ - \tau /{\hat{\mathrm{o}}}}} \right) + b_\theta,$$

where *σ*^2^ is the net MSD and $\tau /{\hat{\mathrm{o}}}$ is a constant from which the microscopic diffusion coefficient can be derived^74^. An intercept ($b_\theta$) was also included and set to the experimental value for MSD($t = 0.0067\;{\mathrm{s}}$) as an estimate of the measurement error inherent in the assay^26^. The fit to the constrained motion equation for the pooled MSD curves is shown as an inset in each panel of supplemental Figs. 7–10.

### Spring constant estimation

The spring constant (*κ*) for the centromere-spring was calculated as follows^25^:

5 $$\kappa = \frac{{k_{\mathrm{b}}T}}{{\sigma ^2}},$$

where *k*_b_ is the Boltzmann constant and *T* is absolute temperature in Kelvin. Spring constant values listed in the text and figures are identified as either *κ* for a spring constant calculated from a MSD curve from an individual chromosome, or $\bar \kappa$ for the mean spring constant calculated from the pooled MSD data for a group of chromosomes.

Standard error for the spring constant (SE_*k*_) was calculated by propagating the standard error of the fitted parameter estimate (${\mathrm{{SE}}}_{\sigma ^2}$) with the measurement error for temperature maintenance based on the heating unit manufacturer’s guidelines (±0.2 °C):

6 $${\mathrm{{SE}}}_\kappa = \left| \kappa \right|\sqrt {\left( {\frac{{k_{\mathrm{b}} \cdot {\mathrm{{SE}}}_T}}{{k_{\mathrm{b}}T}}} \right)^2 + \left( {\frac{{{\mathrm{{SE}}}_{\left\langle {\sigma ^2} \right\rangle }}}{{\left\langle {\sigma ^2} \right\rangle }}} \right)^2}.$$

The relative change in the mean spring constant for the centromere-spring between mitotic stages was expressed as a percent change:

7 $$\Delta \bar \kappa = \left( {\frac{{\bar \kappa _2}}{{\bar \kappa _1}} - 1} \right)100,$$

where $\bar \kappa _1$ is the mean spring constant at the earlier mitotic stage, and $\bar \kappa _2$ is the mean spring constant at the later mitotic stage. The standard error for this estimated was calculated by propagating the standard error from the spring constant estimates:

8 $${\mathrm{{SE}}} = 100\left| {\frac{{\bar \kappa _1}}{{\bar \kappa _2}}} \right|\sqrt {\left( {\frac{{{\mathrm{{SE}}}_1}}{{\bar \kappa _1}}} \right)^2 + \left( {\frac{{{\mathrm{{SE}}}_2}}{{\bar \kappa _2}}} \right)^2}.$$

Linear regression models were used to test the relationship between displacement and spring constant for the centromere-spring at each mitotic stage. Within each stage, chromosomes were subdivided on the basis of their individual sister centromere displacement in intervals of 100 nm, and then the median displacement and mean spring constant were calculated for each group. In the linear regression model, the subgroups’ median displacement and mean spring constant were used as the independent and dependent variables, respectively.

### Centromere force estimation

By modeling the spring-like behavior of the sister centromere linkage (i.e., the centromere-spring) as a Hookean spring, centromere force (*F*_C_) can be defined using the equation for Hooke’s law^25^:

9 $$F_{\mathrm{C}} = \kappa d,$$

where *κ* is the spring constant for the centromere-spring and *d* is displacement of the centromere-spring above its rest length (Eq. (1)). For a detailed discussion of the theory underlying this approach and the supporting rationale, see ref. ^26^.

For an individual chromosome proceeding unperturbed through mitosis at a stage where there was a statistically significant, linear increase in the estimated spring constant as a function of increasing centromere-spring displacement values, the spring constant value was calculated as

10 $$\kappa _{ms,d} = \beta _{0.ms} + (\beta _{ms,d}d),$$

where $\beta _{0,\;ms}$ is the intercept and $\beta _{ms,d}$ is the coefficient for the displacement predictor variable from the linear regression model corresponding to the chromosome’s mitotic stage. In all other cases, the spring constant value used was the mean spring constant for all chromosomes at that stage ($\bar \kappa$).

To compare the potential range in force signal for an individual chromosome across the different mitotic stages, centromere force was calculated over the range of observed centromere-spring displacement values at each stage. The total dynamic range in force signaling for a chromosome at a given stage (DR_*ms*_) was then calculated by subtracting the centromere force magnitude at the minimum observed displacement (*d*_MIN_) from the force value at the maximum observed displacement (*d*_MAX_):

11 $${\mathrm{{DR}}}_{ms} = F_{{\mathrm{C}},ms}\left( {d_{{\mathrm{{MAX}}}}} \right) - F_{{\mathrm{C}},ms}\left( {d_{{\mathrm{{MIN}}}}} \right).$$

### Statistical analysis

All *p* values were two-sided, and *p* < 0.05 was used to indicate statistical significance unless otherwise noted. All probability density functions were calculated using a kernel smoothing function, unless specified otherwise. For parameters with non-Gaussian distributions the median and interquartile range were used to summarize the central tendency and variability in the sample. One-way Kruskal–Wallis tests were used to compare group medians, and the Brown–Forsythe test was used to compared the equality of group variances. Two-sample Kolmogorov–Smirnov tests were used to test for differences in probability distributions. To test for differences between mean spring constant estimates and percent change in spring constants, *z*-tests were performed using the calculated spring constant and the fitted parameter estimate (${\mathrm{{SE}}}_{\sigma ^2}$). To test the relationship between chromosome number and stiffness in the RPE-1-GI cell line, a nonlinear least-squares regression model with an exponential fit equation was used with the group median chromosome number as the independent variable and fold stiffness change as the dependent variable. Cochran–Mantel–Haenszel tests were used to test for a linear trend in the proportion of cells with mitotic defects across groups stratified by chromosome number. Risk for mitotic defects among RPE-1-GI cells was assessed by calculating a relative with the diploid cells as the unexposed group and the aneuploid cells as the exposed group. Standard error for risk ratio was calculated as the standard error of the log relative risk. Results are summarized in the text and figures by the *p* value and relevant test statistic: *z* (*Z*-test), KS_STAT_ (Kolmogorov–Smirnov), BF_STAT_ (Brown–Forsythe), KW_STAT_ (Kruskal–Wallis), *R*^2^ (linear regression), *R*^2^ or *RMSE* (exponential regression), and CMH_Stat_ (Cochran–Mantel-Haenszel). All experiments were performed across a minimum of three unique experimental days.

## Supplementary information

Supplementary Information

Source Data

## Data Availability

The data that support the findings of this study are available from the corresponding author upon request.

## Code availability

The custom computer code that supports the findings of this study are available from the corresponding author upon request.
