# Supplementary material for: Centromere mechanical maturation during mammalian cell mitosis
Source: Nat Commun. 2019 Apr 15;10:1761. doi: 10.1038/s41467-019-09578-z (PMC6465287; doi:10.1038/s41467-019-09578-z)
Supplement: Supplementary file 1 — Supplementary Information [file 41467_2019_9578_MOESM1_ESM.pdf]

Supplementary Material

Harasymiw et al, Centromere mechanical maturation during mammalian cell mitosis

Supplementary Figure 1………………………..Page 2

Supplementary Figure 2………………………..Page 5

Supplementary Figure 3………………………..Page 7

Supplementary Figure 4………………………..Page 9

Supplementary Figure 5………………………..Page 11

Supplementary Figure 6………………………..Page 13

Supplementary Figure 7………………………..Page 15

Supplementary Figure 1

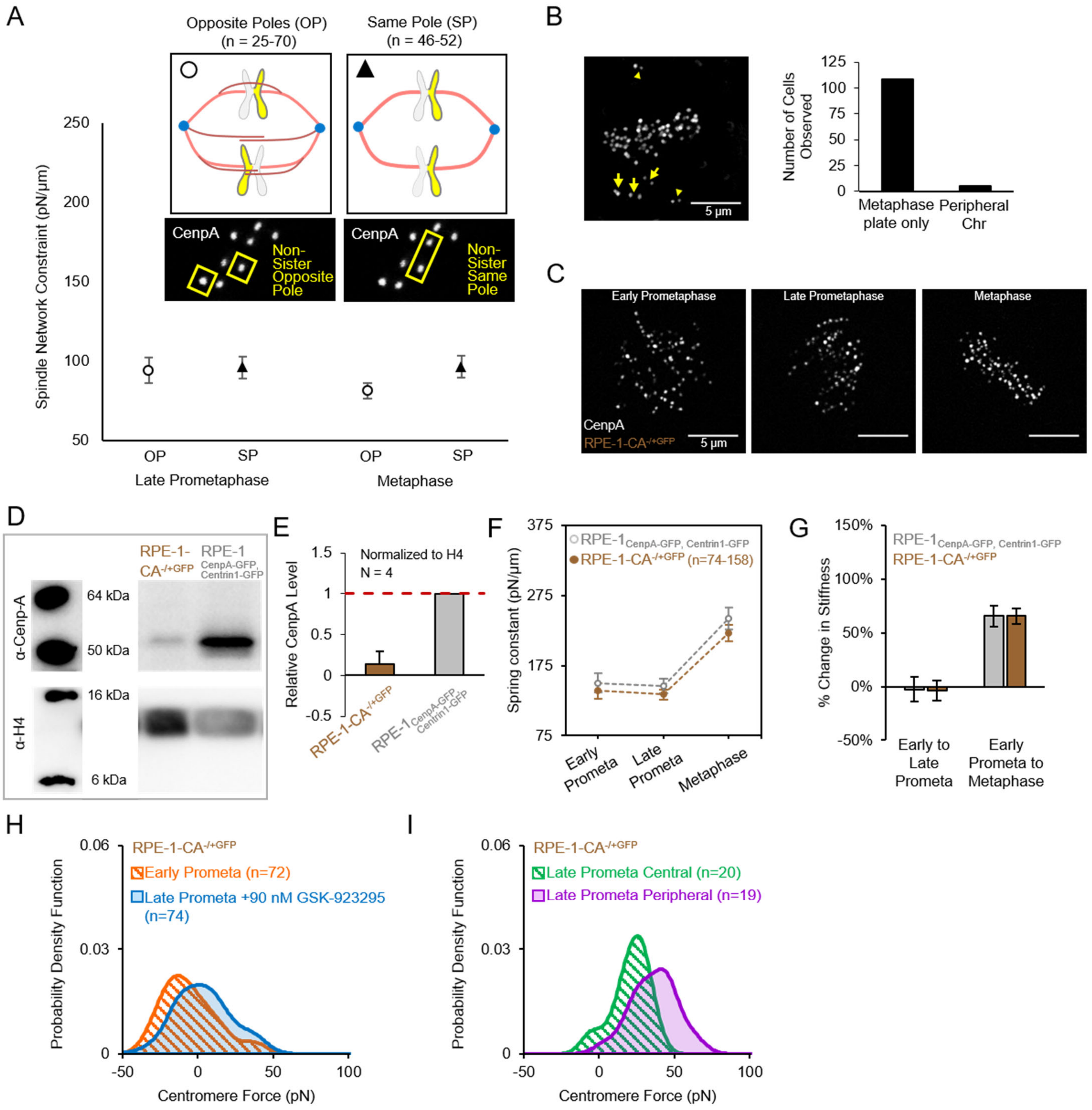

Supplementary Figure 1. Validation data for measuring the stiffness of the centromere-spring in human cells.

(A) To ask whether bridging fibers and crosslinking proteins associated with interpolar microtubule overlap (eg, pole-to-pole connections) led to significant spindle stiffening from late prometaphase to metaphase, we analyzed the motion of the CenpA-GFP tags between non-sister centromeres that were attached to opposite poles (yellow, top-left), as assessed by their orientation in the spindle (middle-left). Here, we evaluated the motions of opposite-pole-attached non-sisters (yellow, top-left), which were constrained by bridging fibers and interpolar microtubule overlaps (dark red, top-left). We found that for the opposite-pole-attached non-sister centromeres (OP), there was no significant increase in spindle network centromere constraint from late prometaphase to metaphase (bottom, open circles), indicating that centromere mechanical maturation was not driven by a maturation of opposite-pole-attached spindle network constraints, but rather by maturation of the centromeric chromatin itself. Second, to ask whether spindle pole compliances (eg, pole-to-microtubule connections) led to significant spindle stiffening from late prometaphase to metaphase, we analyzed the motion of the CenpA-GFP tags between non-sister centromeres that were attached to the same pole (yellow, top-right), as assessed by their orientation in the spindle (middle-right). Here, we evaluated the motions of same-pole-attached non-sisters (yellow, top-right), which were constrained by the connection between kinetochore fibers (red, top-right) and the spindle poles (blue circle, top-right). We found that for the same-pole-attached non-sister centromeres (SP), there was no significant increase in spindle network centromere constraint from late prometaphase to metaphase (bottom, closed triangles), indicating that, similar to the opposite-pole-attached results, centromere mechanical maturation was not driven by a maturation of same-pole-attached spindle network constraints, but rather by maturation of the centromeric chromatin itself. Error bars represent standard error.

(B) *Left:* Live cell imaging of an RPE-1 cell expressing CenpA-GFP at the outer centromere and centrin1-GFP at the spindle poles. Image is a maximum intensity projection from a full-cell-volume image series, and is representative of cells observed with both a clearly formed metaphase plate and peripheral chromosomes. Yellow arrowheads indicated Centrin1 at the spindle poles. Yellow arrows indicate centromere pairs for chromosomes located off the metaphase plate in the cell's periphery. Scale bar, 5 microns. *Right:* Frequency of cells with formed metaphase plates only versus those with both a plate and peripheral chromosomes. Data was pooled from 3 independent experiments.

(C) Live cell imaging of RPE-1-CA$^{-/+GFP}$ cells expressing CenpA-GFP at the outer centromere. In contrast with the RPE-1$_{CenpA\text{-}GFP,Centrin1\text{-}GFP}$ cell line, which expresses CenpA-GFP from an exogenous, constitutively-active CMV promoter on a stably-integrated plasmid, the RPE-1-CA$^{-/+GFP}$ cell line was constructed by knocking out an essential portion of the CenpA gene at one native locus, and then inserting a knock-in allele with GFP encoding sequences in frame with the CenpA gene at the alternate locus. Images are maximum intensity projections from full-cell-volume image series, and are representative of the described mitotic stages: early-prometaphase (left), late-prometaphase (center) and metaphase (right). Scale bar, 5 microns.

(D) Western blot for CenpA and Histone 4 (H4) in RPE-1-CA$^{-/+GFP}$ and RPE-1$_{CenpA\text{-}GFP,Centrin1\text{-}GFP}$ cells. Image is from one of four independent experiments.

(E) Quantification of CenpA levels normalized to their respective H4 levels in RPE-1-CA$^{-/+GFP}$ cells and RPE-1$_{CenpA\text{-}GFP,Centrin1\text{-}GFP}$ cells, and then data from both cells normalized to expression in the RPE-1$_{CenpA\text{-}GFP,Centrin1\text{-}GFP}$ cells. Sample size (N) represents number of independent experiments.

(F-G) Mean spring constant by mitotic stage for RPE-1-CA$^{-/+GFP}$ cells (I, brown filled data points) and the relative change in the centromere-spring's stiffness across mitotic stages (J, brown bars). Data from RPE-1$_{CenpA\text{-}GFP,Centrin\text{-}1}$ cells included for comparison (I, grey unfilled data points; J, grey bars). Error bars represent standard error. Note that the relative change in stiffness is highly comparable between the two cell types.

(H-I) Probability density functions for centromere force (F_C) in (K) early-prometaphase chromosomes (orange line, patterned fill) versus GSK-923295-treated late-prometaphase chromosomes (blue line, shaded fill) and (L) central late-prometaphase chromosomes (green line, patterned fill) versus peripheral late-prometaphase chromosomes (purple line, shaded fill).

Abbreviations: Prometaphase (prometa), mean squared displacement (MSD), histone 4 (H4) chromosome (Chr). Sample sizes (n) reflect the number of chromosomes in each group or distribution; for panels with multiple groups the range in number of chromosomes per group is presented (smallest group-largest group). Sample sizes (N) reflect the number of independent experiments.

Supplementary Figure 2

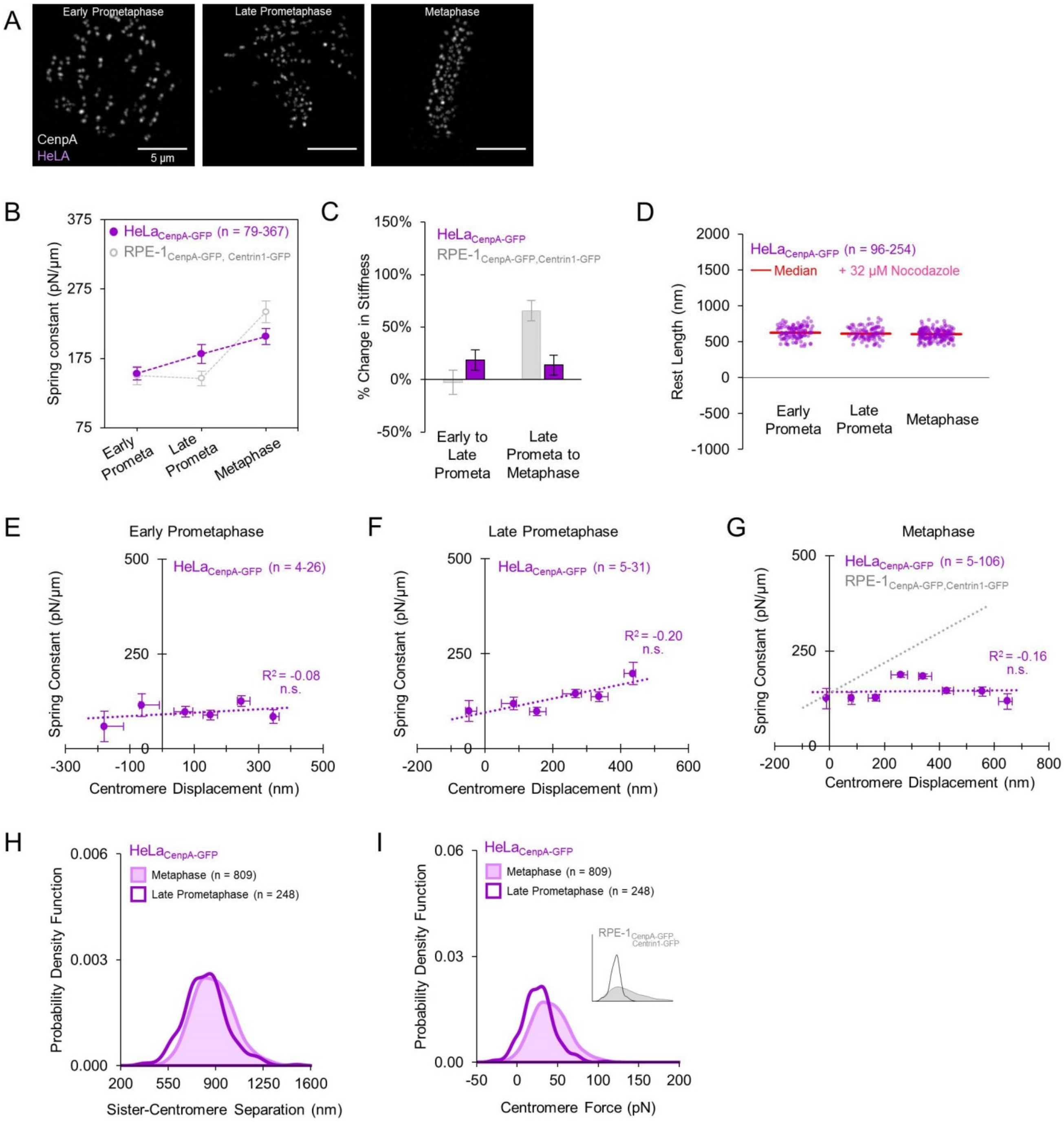

Supplementary Figure 2. Characterization of centromere mechanics in adenocarcinoma HeLa cells.

(A) Live cell imaging of HeLa cells expressing CenpA-GFP at the outer centromere. Images are maximum intensity projections from full-cell-volume image series, and are representative of the described mitotic stages: early-prometaphase (left), late-prometaphase (center) and metaphase (right). Scale bar, 5 microns.

(B-C) Mean spring constant by mitotic stage for HeLa cells (B, purple filled data points) and the relative change in the stiffness of the centromere-spring across mitotic stages (C, purple bars). Data from RPE-1 cells included for comparison (B, grey unfilled data points; C, grey bars). Error bars represent standard error.

(D) Quantification of the rest length of the centromere-spring (l_R), by mitotic stage for HeLa cells. Data points reflect individual chromosomes pooled across three independent experiments, the group median is shown in red.

(E-G) Relationship between displacement of the centromere-spring and its spring constant at early-prometaphase (E), late-prometaphase (F), and metaphase (G) for HeLa cells. Chromosomes were sub-grouped by displacement in 100 nm intervals starting at the group minimum. Only subgroups with 4 or more chromosomes are shown. Each data point reflects the subgroup's median displacement and mean spring constant; X-axis error bars represent IQR, Y-axis error bars represent standard error. The least-squares regression fit is indicated by the dotted-line and the listed R^2 value. Exact p-values are shown for models meeting statistical significance; all others are indicated as non-significant (n.s.). The least-squares regression fit line for RPE-1 chromosomes at metaphase is shown for comparison (G, dotted grey line).

(H-I) Probability density functions for (H) sister centromere separation (s) and (I) centromere force (F_C) (I) at late-prometaphase (dark purple line) and metaphase (light purple line, shaded area) in the HeLa cells. Note the near overlap in distributions for both separation and force. *Inset* (I): Probability density function for RPE-1 centromere force at late-prometaphase (black line) and metaphase (grey line, shaded area) are shown for comparison.

Abbreviations: Prometaphase (prometa), interquartile range (IQR). Sample sizes (n) reflect the number of chromosomes in each group or distribution; for panels with multiple groups the range in number of chromosomes per group is presented (smallest group-largest group). See also Figure S7.

Supplementary Figure 3

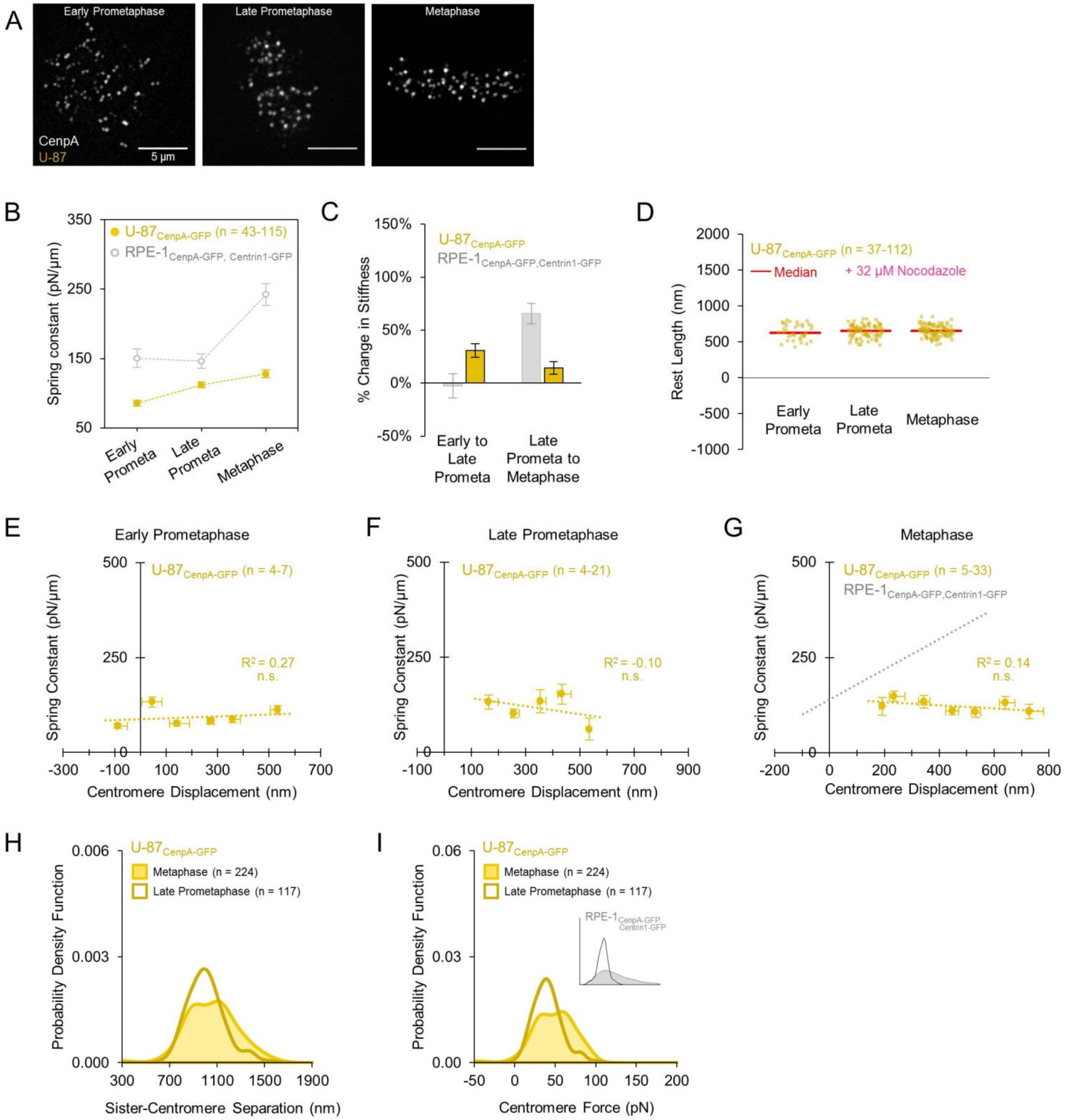

Supplementary Figure 3. Characterization of centromere mechanics in glioblastoma U-87 cells.

(A) Live cell imaging of U-87 cells expressing CenpA-GFP at the outer centromere. Images are maximum intensity projections from full-cell-volume image series, and are representative of the described mitotic stages: early-prometaphase (left), late-prometaphase (center) and metaphase (right). Scale bar, 5 microns.

(B-C) Mean spring constant by mitotic stage for U-87 cells (B, gold filled data points) and the relative change in the stiffness of the centromere-spring across mitotic stages (C, gold bars). Data from RPE-1 cells included for comparison (B, grey unfilled data points; C, grey bars). Error bars represent standard error.

(D) Quantification of the rest length of the centromere-spring (l_R), by mitotic stage for the U-87 cells. Data points reflect individual chromosomes pooled across three independent experiments, the group median is shown in red.

(E-G) Relationship between the displacement of the centromere-spring and its spring constant at early-prometaphase (E), late-prometaphase (F), and metaphase (G) in the U-87 cells. Chromosomes were sub-grouped by displacement in 100 nm intervals starting at the group minimum. Only subgroups with 4 or more chromosomes are shown. Each data point reflects the subgroup's median displacement and mean spring constant; X-axis error bars represent IQR, Y-axis error bars represent standard error. The least-squares regression fit is indicated by the dotted-line and the listed R^2 value. Exact p-values are shown for models meeting statistical significance; all others are indicated as non-significant (n.s.). The least-squares regression fit line for RPE-1 chromosomes at metaphase is shown for comparison (G, dotted grey line).

(H-I) Probability density functions for (H) sister centromere separation (s) and (I) centromere force (F_C) at late-prometaphase (dark gold line) and metaphase (light gold line, shaded area) in the U-87 cells. Note the near overlap in distributions for both separation and force. *Inset* (I): RPE-1 centromere force distributions at late-prometaphase (black line) and metaphase (grey line, shaded area) are shown for comparison.

Abbreviations: Prometaphase (prometa), interquartile range (IQR). Sample sizes (n) reflect the number of chromosomes in each group or distribution; for panels with multiple groups the range in number of chromosomes per group is presented (smallest group-largest group). See also Figure S7.

Supplementary Figure 4

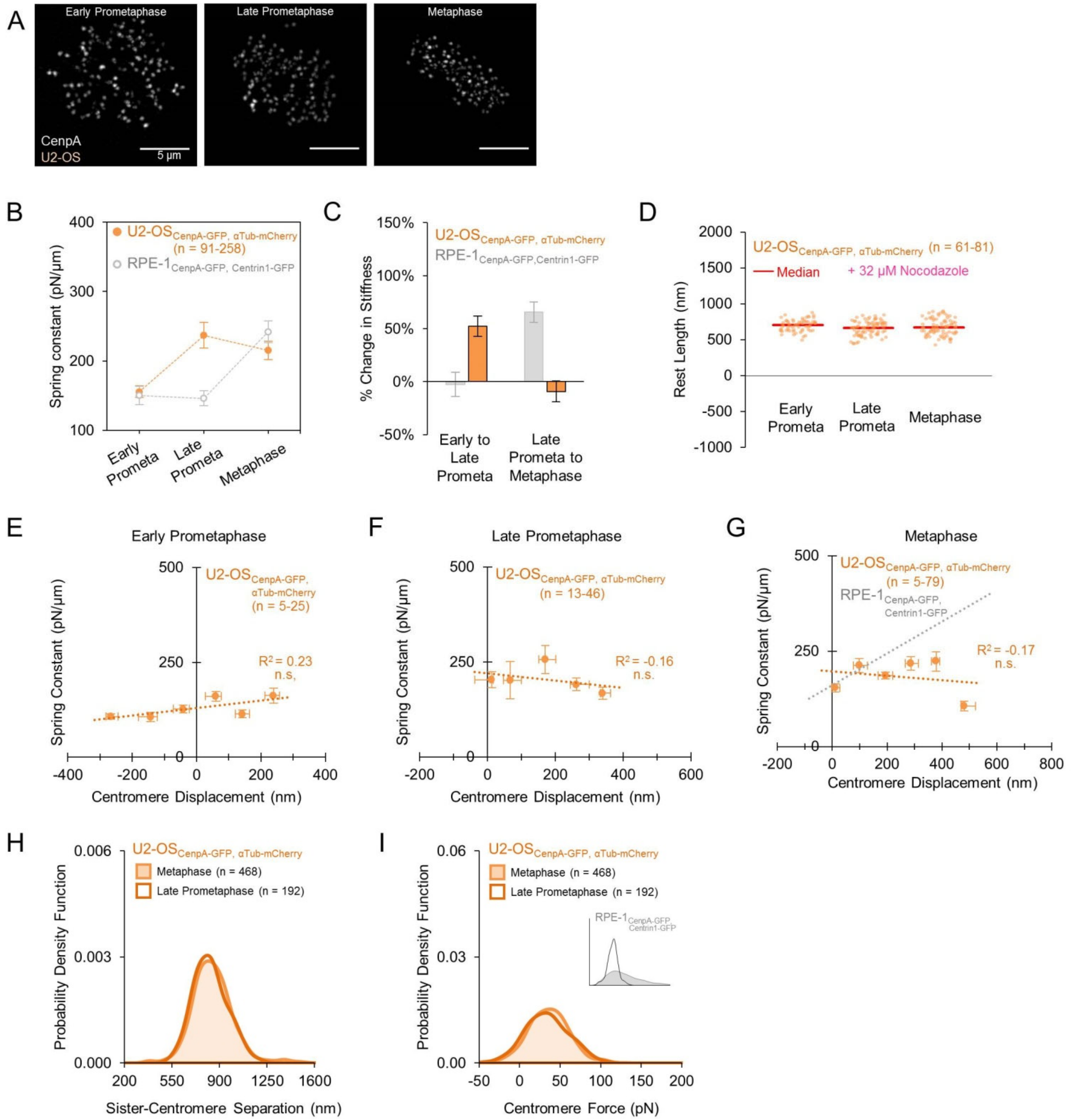

Supplementary Figure 4. Characterization of centromere mechanics in osteosarcoma U2-OS cells.

(A) Live cell imaging of U2-OS cells expressing CenpA-GFP at the outer centromere. Images are maximum intensity projections from full-cell-volume image series, and are representative of the described mitotic stages: early-prometaphase (left), late-prometaphase (center) and metaphase (right). Scale bar, 5 microns.

(B-C) Mean spring constant by mitotic stage for U2-OS cells (B, orange filled data points) and the relative change in the stiffness of the centromere-spring across mitotic stages (C, orange bars). Data from RPE-1 cells included for comparison (B, grey unfilled data points; C, grey bars). Error bars represent standard error.

(D) Quantification of the rest length of the centromere-spring (l_R), by mitotic stage in the U2-OS cells. Data points reflect individual chromosomes pooled across three independent experiments, the group median is shown in red.

(E-G) Relationship between the displacement of the centromere-spring and its spring constant at early-prometaphase (E), late-prometaphase (F), and metaphase (G) in the U2-OS cells. Chromosomes were sub-grouped by displacement in 100 nm intervals starting at the group minimum. Only subgroups with 4 or more chromosomes are shown. Each data point reflects the subgroup's median displacement and mean spring constant; X-axis error bars represent IQR, Y-axis error bars represent standard error. The least-squares regression fit is indicated by the dotted-line and the listed R^2 value. Exact p-values are shown for models meeting statistical significance; all others are indicated as non-significant (n.s.). The least-squares regression fit line for RPE-1 chromosomes at metaphase is shown for comparison (G, dotted grey line).

(H-I) Probability density functions for (H) sister centromere separation (s) and (I) centromere force (F_C) at late-prometaphase (dark orange line) and metaphase (light orange line, shaded area) in the U2-OS cells. Note the near overlap in distributions for both separation and force. *Inset* (I): RPE-1 centromere force distributions at late-prometaphase (black line) and metaphase (grey line, shaded area) are shown for comparison.

Abbreviations: Prometaphase (prometa), interquartile range (IQR). Sample sizes (n) reflect the number of chromosomes in each group or distribution; for panels with multiple groups the range in number of chromosomes per group is presented (smallest group-largest group). See also Figure S7.

Supplementary Figure 5

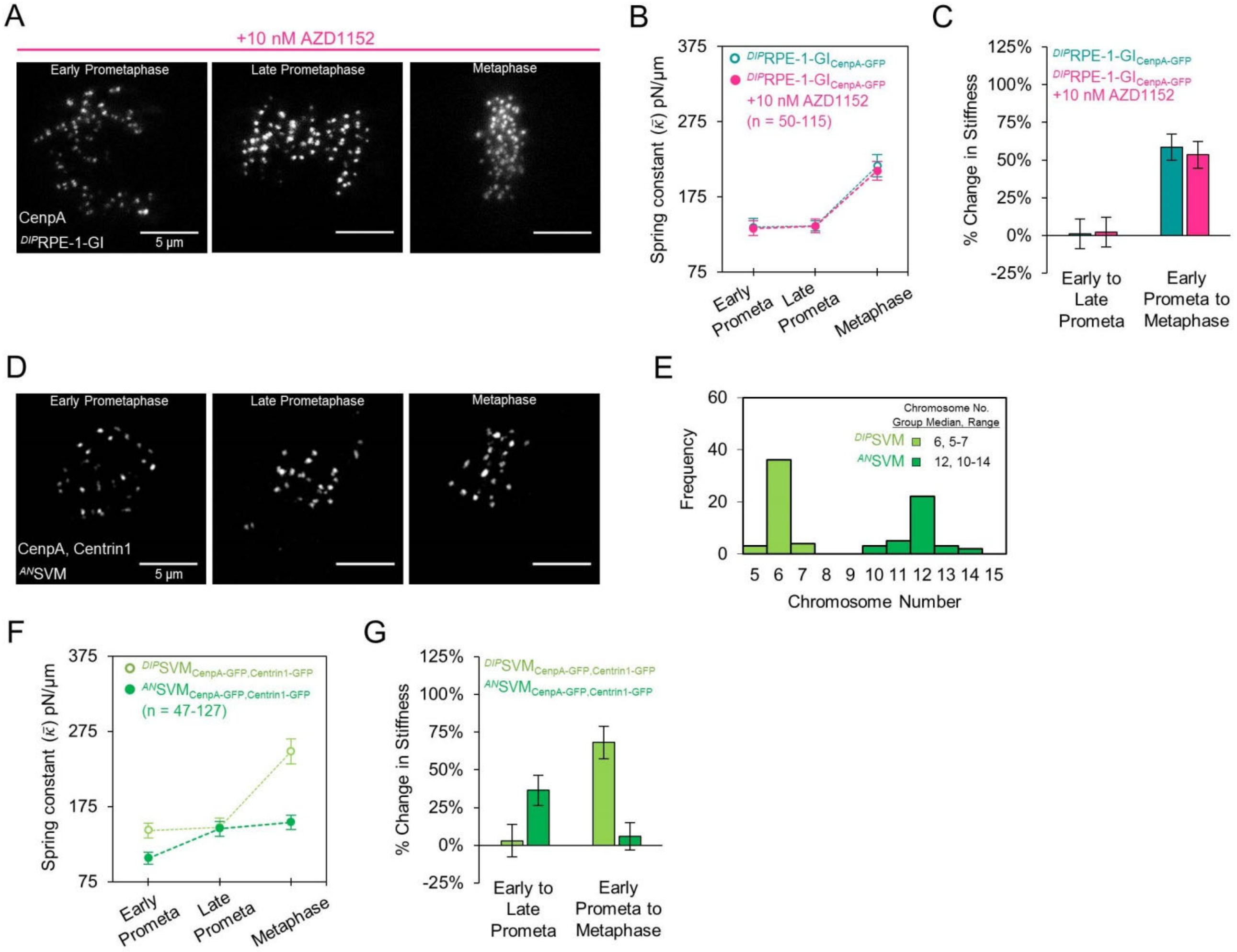

Supplementary Figure 5. Characterization of centromere mechanics in aneuploid SVM-muntjac cells.

(A) Live cell imaging of ^{DIP}RPE-1-GI cells expressing CenpA-GFP at the outer centromere and treated with 10 nM AZD-1152, a kinase inhibitor that is highly selective for Aurora-B. Images are maximum intensity projections from full-cell-volume image series, and are representative of the described mitotic stages: early prometaphase (left), late prometaphase (center) and metaphase (right).

(B-C) Mean spring constant by mitotic stage for ^{DIP}RPE-1-GI cells treated with 10 nM AZD-1152 (B, pink filled data points), and the relative change in the centromere-spring's stiffness across mitotic stages (C, pink bars). Data from untreated ^{DIP}RPE-1-GI cells included for comparison (B, teal unfilled data points; F, teal bars). Note that both the absolute spring constant estimates and the relative change in stiffness are highly comparable between the two cell types.

(D) Live cell imaging of muntjac ^{AN}SVM cells expressing CenpA-GFP at the outer centromere and centrin1-GFP at the spindle poles. Images are maximum intensity projections from full-cell-volume image series, and are representative of the described mitotic stages: early-prometaphase (left), late-prometaphase (center) and metaphase (right).

(E) Histogram of chromosome numbers for a sample of SVM cells differentiating between diploid cells (^{DIP}SVM; light green bars, median = 6, mode = 6, range = 5-7) and aneuploid cells (^{AN}SVM; dark green bars, median = 12, mode = 12, range = 10-14).

(F-G) Mean spring constant by mitotic stage for ^{AN}SVM cells (C, dark green filled data points) and the relative change in the centromere-spring's stiffness across mitotic stages (D, dark green bars). Data from ^{DIP}SVM cells included for comparison (K,light green unfilled data points; L, light green bars). Error bars represent standard error.

Abbreviations: Aneuploid (AN), diploid (DIP), prometaphase (prometa). Sample sizes (n) reflect the number of chromosomes in each group or distribution; for panels with multiple groups the range in number of chromosomes per group is presented (smallest group-largest group). See also Figure S6.

Supplementary Figure 6

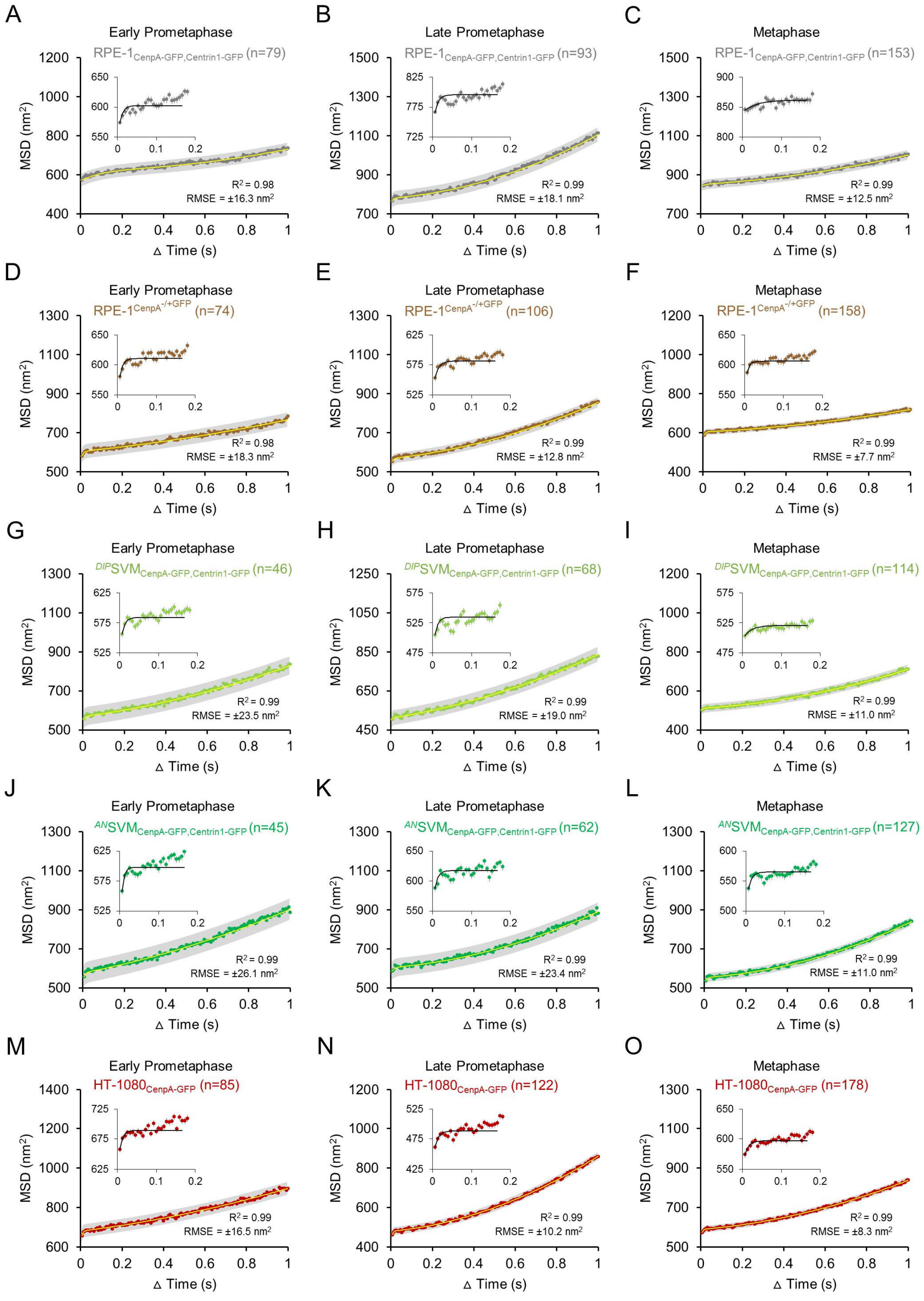

Supplementary Figure 6. Pooled mean squared displacement curves for RPE-1, SVM and HT-1080 cell lines.

(A-O) Pooled mean squared displacement (MSD) curves by mitotic stage for RPE-1$_{\text{CenpA-GFP,Centrin-1}}$ (A-C), RPE-1$^{\text{CenpA-/+GFP}}$ (D-F), ^{DIP}SVM$_{\text{CenpA-GFP,Centrin-1}}$ (G-I), ^{AN}SVM$_{\text{CenpA-GFP,Centrin-1}}$ (J-L), and HT-1080 (M-O) cells. Data points represent the mean squared displacement at each time interval averaged across all chromosomes at the specified mitotic stage. The results of the nonlinear regression fit to the MSD mixed-motion equation are indicated by the yellow fit line and the grey 95% confidence region, as well as the indicated R^2 and $RMSE$ values. Error bars represent standard error. Sample sizes (n) reflect the number of chromosomes in each group. *Insets*: MSD data over the timescale for thermal fluctuations. Fit line for the MSD constrained motion model is shown in black.

Supplementary Figure 7

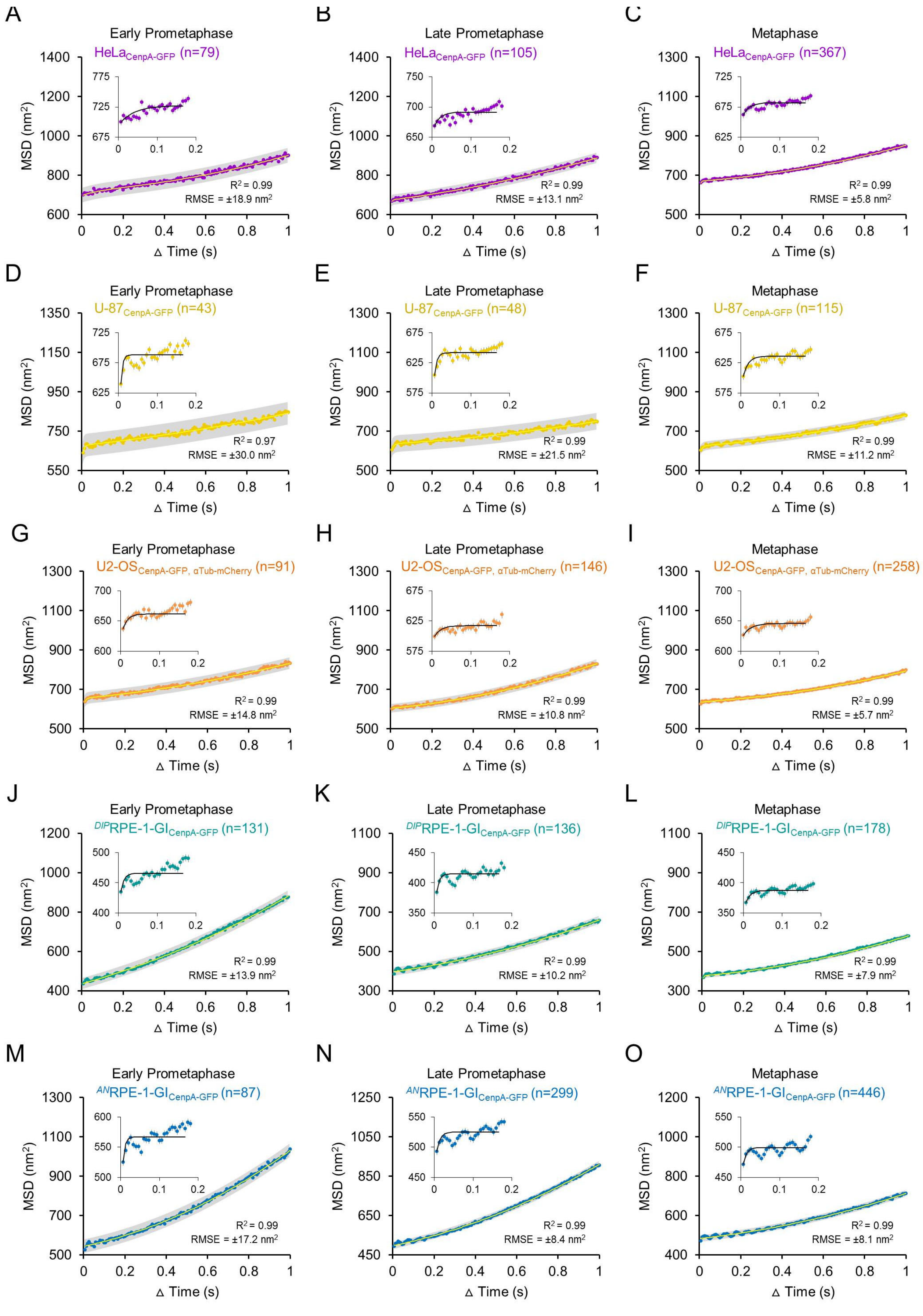

Supplementary Figure 7. Pooled mean squared displacement curves for HeLa, U-87, U2-OS, and RPE-1-GI cell lines.

(A-O) Pooled mean squared displacement curves by mitotic stage for HeLa (A-C), U-87 (D-F), U2-OS(G-I), ^{DIP}RPE-1-GI (J-L), and ^{AN}RPE-1-GI (M-O) cells. Data points represent the mean squared displacement at each time interval averaged across all chromosomes at the specified mitotic stage. The results of the nonlinear regression fit to the MSD mixed-motion equation are indicated by the yellow fit line and the grey 95% confidence region, as well as the indicated R^2 and $RMSE$ values. Error bars represent standard error. Sample sizes (n) reflect the number of chromosomes in each group. *Insets*: MSD data over the timescale for thermal fluctuations. Fit line for the MSD constrained motion model is shown in black.
