# Supplementary figures and images for: Centromere mechanical maturation during mammalian cell mitosis
Source: Nat Commun. 2019 Apr 15;10:1761. doi: 10.1038/s41467-019-09578-z (PMC6465287; doi:10.1038/s41467-019-09578-z)

Anti-CENPA Antibody

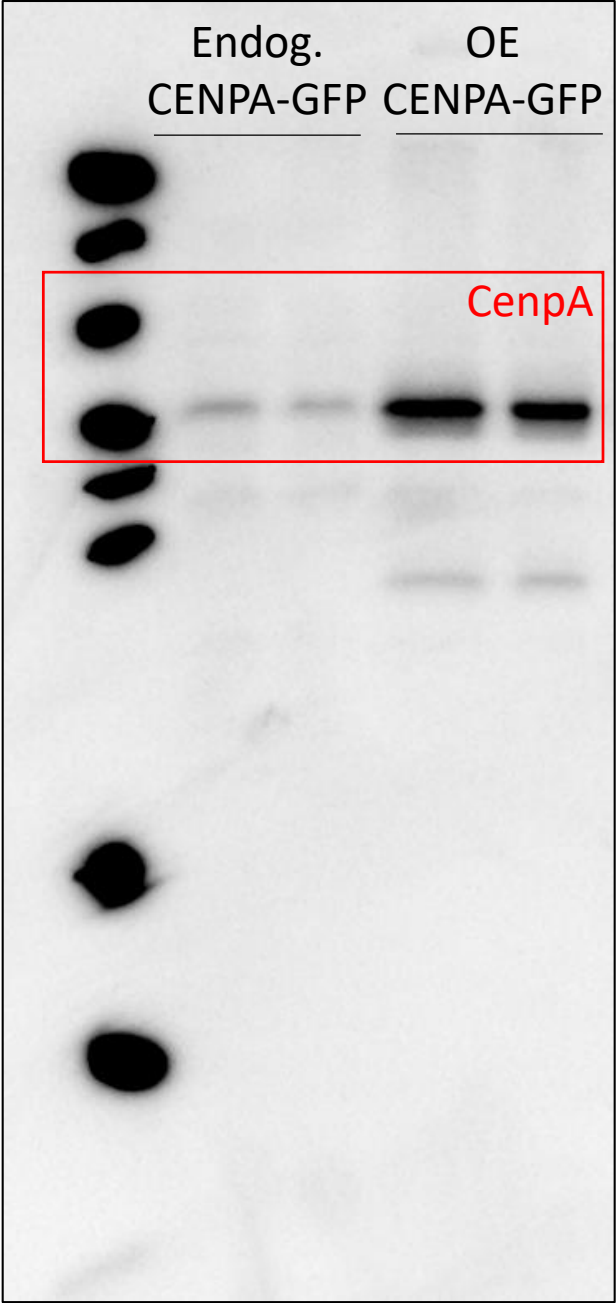

Anti-H4 Antibody

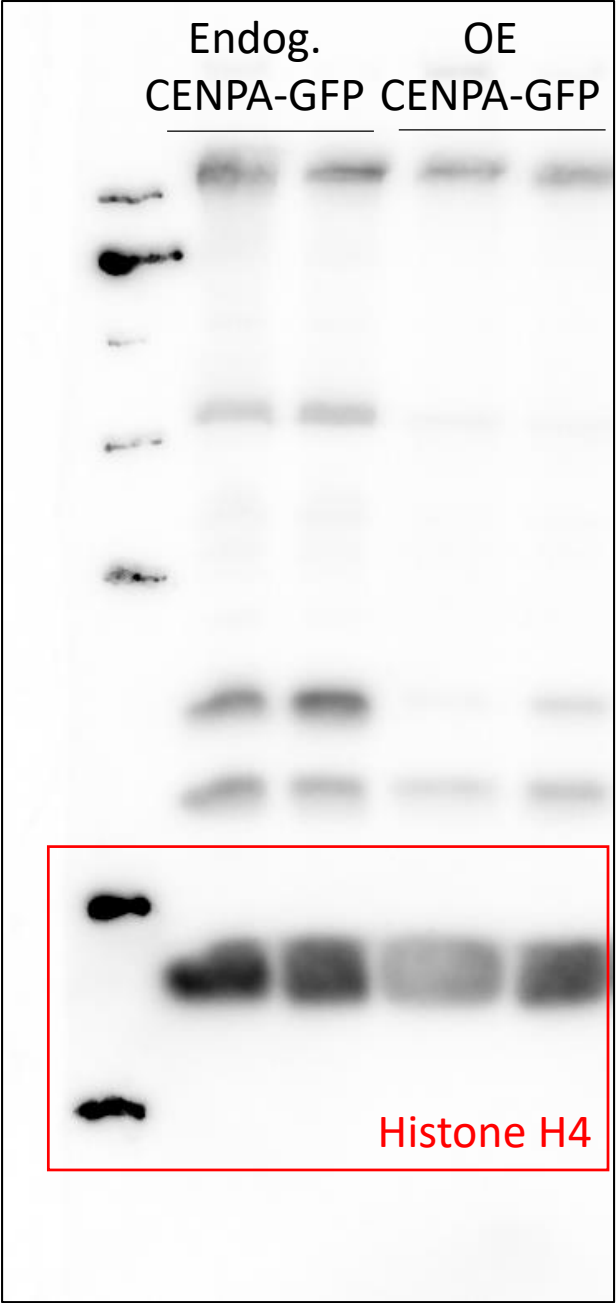

Supplement: Supplementary file 2 — Source Data [file 41467_2019_9578_MOESM2_ESM.zip › Source Data.pdf]
